# Synthesis of Rhodamine-Conjugated Lupane Type Triterpenes of Enhanced Cytotoxicity

**DOI:** 10.3390/molecules29102346

**Published:** 2024-05-16

**Authors:** Toni C. Denner, Niels V. Heise, Sophie Hoenke, René Csuk

**Affiliations:** Organic Chemistry, Martin-Luther University Halle-Wittenberg, Kurt-Mothes, Str. 2, 06120 Halle (Saale), Germany

**Keywords:** betulinic acid, platanic acid, rhodamine hybrids, cytotoxicity

## Abstract

Various conjugates with rhodamines were prepared by starting with betulinic acid (**BA**) and platanic acid (**PA**). The molecules homopiperazine and piperazine, which were identified in earlier research, served as linkers between the rhodamine and the triterpene. The pentacyclic triterpene’s ring A was modified with two acetyloxy groups in order to possibly boost its cytotoxic activity. The SRB assays’ cytotoxicity data showed that conjugates **13**–**22**, derived from betulinic acid, had a significantly higher cytotoxicity. Of these hybrids, derivatives **19** (containing rhodamine B) and **22** (containing rhodamine 101) showed the best values with EC_50_ = 0.016 and 0.019 μM for A2780 ovarian carcinoma cells. Additionally, based on the ratio of EC_50_ values, these two compounds demonstrated the strongest selectivity between malignant A2780 cells and non-malignant NIH 3T3 fibroblasts. A375 melanoma cells were used in cell cycle investigations, which showed that the cells were halted in the G1/G0 phase. Annexin V/FITC/PI staining demonstrated that the tumor cells were affected by both necrosis and apoptosis.

## 1. Introduction

The modification of natural products to discover and produce pharmaceutically active compounds remains an important aspect of modern drug research. Thereby, some classes of secondary natural products play an outstanding role, e.g., alkaloids or terpenes [1,2,3,4,5,6,7,8,9,10,11,12,13,14,15,16,17,18,19]. The pentacyclic triterpene betulinic acid (**BA**), for example, was first described in 1995 by E. Pisha [20] regarding its cytotoxic activity, and since then an almost innumerable number of investigations have been carried out on BA, its derivatives, and other triterpenes. Recently, pentacyclic triterpene rhodamine conjugates have been studied more intensively. These compounds are often characterized by a particularly high cytotoxicity, which in some cases lies even in the sub-nanomolar concentration range [21,22,23,24,25,26,27,28,29,30]. At the same time, some of these compounds also showed very good tumor cell/non-tumor cell selectivity, and demonstrated efficacy not only in 2D but also in 3D tumor cell spheroid models [21].

However, it was also shown that the cytotoxic activity of these conjugates depends on all three constituents that build them up: on the one hand, this is the choice of the “right” rhodamine, the suitable spacer between the rhodamine and the triterpene, as well as its type of linkage (amides proved to be better suited than esters; the spacer is preferably a cyclic, secondary amine) and the triterpene itself [25]. Regarding the latter, it was shown that pentacyclic triterpenes exhibit higher cytotoxicity when ring A carries not one but two hydroxyl groups (protected as acetates). Thus, derivatives of maslinic acid were always superior to those of oleanolic acid [31,32,33,34,35,36,37,38,39,40,41,42,43,44,45,46,47], and derivatives of corosolic acid were always better than those of ursolic acid [48,49,50,51,52]. It is therefore obvious to extend these investigations to the field of lupane-type triterpenes and to use differently substituted rhodamines. Since piperazine and homopiperazine spacers have proved particularly successful in the past [25,29], they should also be used in these studies. As an example, we also aimed to investigate how the replacement of the methylene group on betulinic acid with a keto group affects the cytotoxicity of the compounds, and what influence the replacement of an sp^2^-hybridised center on C-20 with an sp^3^-hybridised center has.

## 2. Results

**Figure 1:**
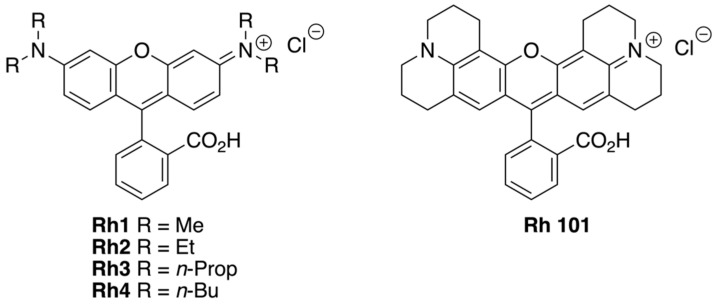
Structure of the rhodamines **Rh1**–**Rh4** and of rhodamine 101 (**Rh101**).

The synthesis of the differently substituted rhodamines **Rh1**–**Rh4** has been described by us before using 3-aminophenol as a starting material [23,33]; their structures as well as those of commercially obtained rhodamine 101 (**Rh101**) are depicted in Figure 1.

**Scheme 1:**
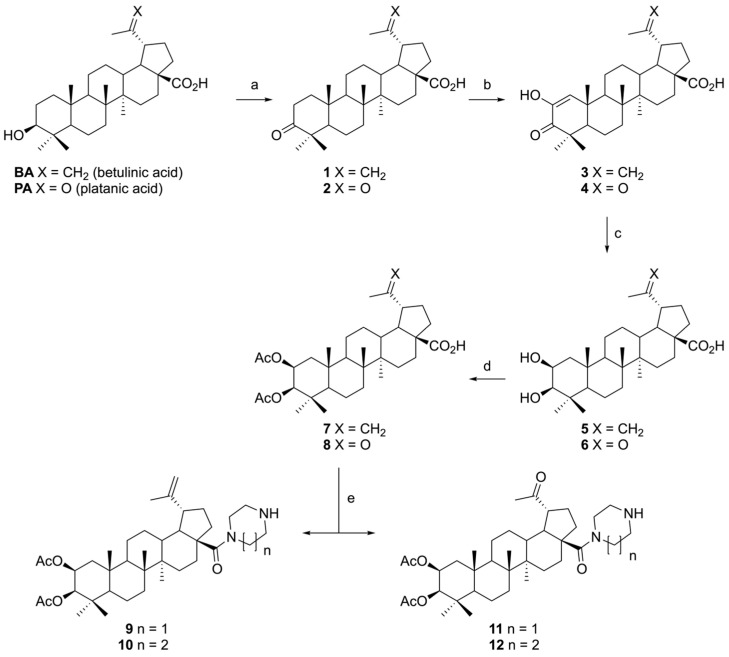
Reactions and conditions for the modification on the lupine framework (a) Jones oxidation, 76% for **1**, 82% for **2**; (b) ^t^BuOK, ^t^BuOH, THF, 3 h, 50 °C, air, 97% for **3**, 75% for **4**; (c) NaBH_4_, THF, MeOH, rt, 1 d, 79% (for **5**), 87% (for **6**); (d) Ac_2_O, NEt_3_, DMAP (cat.), DCM, rt, 1 d, 86% (for **7**), 92% (for **8**); (e) (COCl)_2_, DCM, rt, 30 min, then piperazine or homopiperazine, DCM, 3 h: yields: **9**: 70%; **10**: 85%; **11**: 90%; **12**: 70%.

The synthesis of the conjugates was carried out starting from betulinic acid (**BA**) and platanic acid (**PA**, Scheme 1); the latter differs from **BA** in that the C-30 methylene group is replaced by a keto group. A silica-supported Jones oxidation of **BA** and **PA** gave products **1** and **2** in good yields [53,54,55]. These were reacted with *t*-BuOK/air in *t*-BuOH to produce the C-2 β-configured compounds **3** and **4** [56,57], the reduction of which with NaBH_4_ gave the 2β,3β-configured diols **5** and **6** [55]. Their acetylation [55] with acetic anhydride yielded the acetates **7** and **8** [55]. 

These carboxylic acids were first reacted with oxalylic chloride, and the resulting in situ carboxylic acid chlorides were each treated with either piperazine or homopiperazine; this gave the **BA**-derived amides **9** and **10** and the **PA**-derived amides **11** and **12**.

**Scheme 2:**
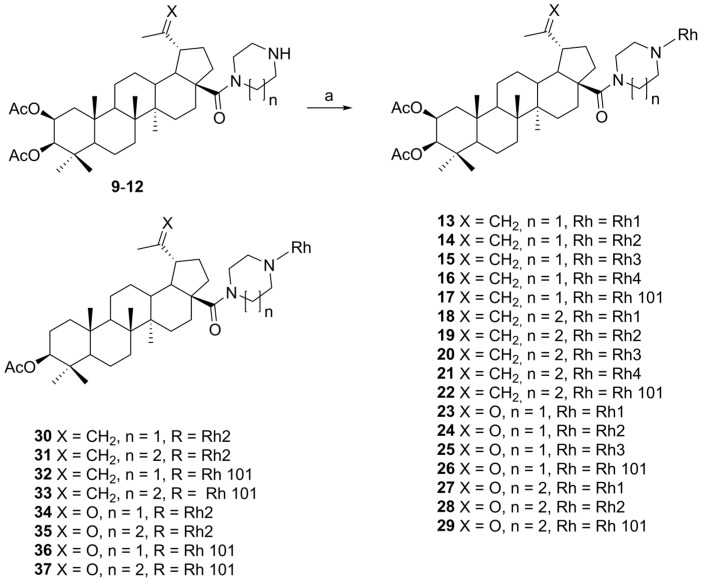
Synthesis of the rhodamine-conjugates holding a lupane skeleton: (a) rhodamines **Rh1**–**Rh4** and **Rh101**, oxalyl chloride, DMF (cat.), DCM, 0 °C → 21 °C, 2 h, then lupane amide, NEt_3_, DMAP (cat.), DCM, 21 °C, 24 h.

The activation of rhodamines **Rh1**–**Rh4** and **Rh101** was also carried out with oxalyl chloride/DMF (cat.); the carboxylic acid chlorides thus generated in situ were reacted with amines **9**–**12** to produce (Scheme 2) the corresponding conjugates **13**–**29**. Thereby, compounds **30**–**37** have been included into this study to investigate the influence of the presence of a second acetyloxy moiety attached to ring A (as in **13**–**29**).

**Scheme 3:**
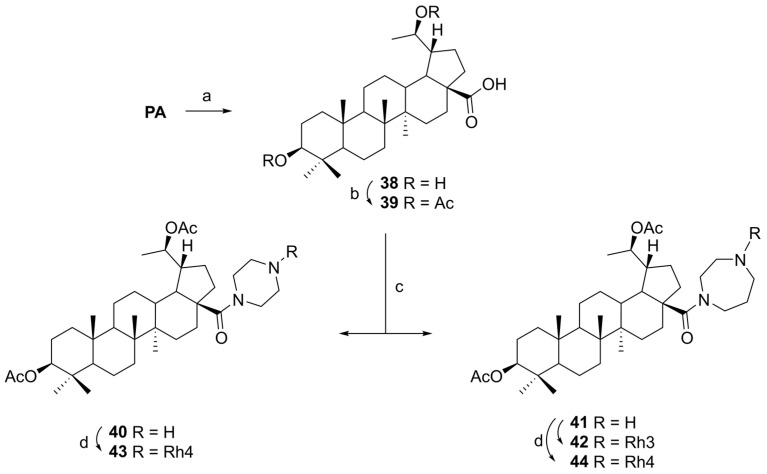
Synthesis of conjugates **38**–**44**; reactions and conditions: (a) NaBH_4_, THF/MeOH, 21 °C, 3 d, 59% of **38**; (b) Ac_2_O, NEt_3_, DMAP (cat.), DCM, 21 °C, 1 d, 86% of **39**; (c) oxalyl chloride, NEt_3_, DMF (cat.), DCM, 21 °C, 30 min, then piperazine (→**40**, 82%) and homopiperazine (→**41**, 72%), DCM, NEt_3_, DMAP, 0 °C → 21 °C, 30 min; (d) rhodamines **3** or **4**, oxalyl chloride, DMF (cat.), DCM, 0 °C → 21 °C, 1 h, then amide, NEt_3_, DMAP (cat.), DCM, 21 °C, 24 h; →**42** (64%), **43** (60%), **44** (62%).

To further investigate the influence of a sp^3^- instead of an sp^2^-hybridized center on the cytotoxicity of the compounds, **38** was prepared from **PA** as previously described, (Scheme 3) followed by its acetylation to yield **39**; this compound was converted into the piperazinyl and homopiperazinyl amides **40** and **41**, respectively. Their coupling with some selected rhodamines led to the conjugates **42**–**44**, respectively.

**Figure 2:**
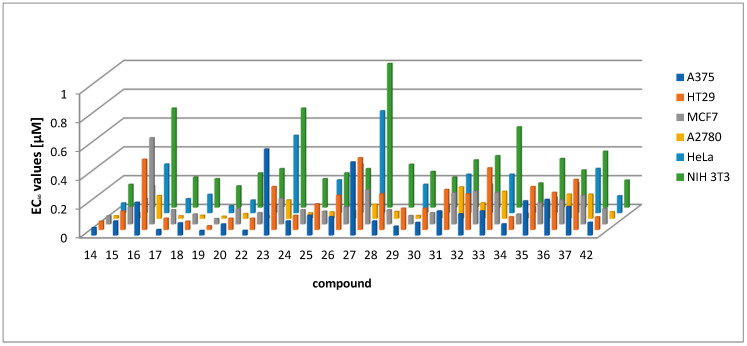
Cytotoxicity of selected compounds; SRB assay EC_50_ values [μM] after 72 h of treatment.

**Figure 3:**
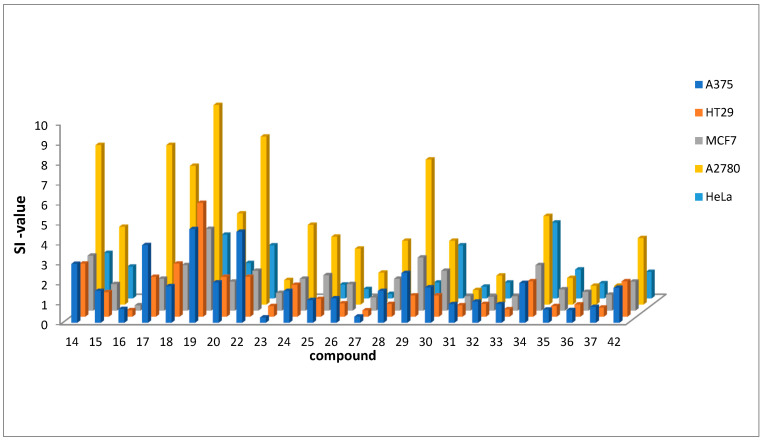
Selectivity S = EC_50_ (NIH 3T3)/EC_50_ (tumor cell line) of selected compounds.

All triterpene rhodamine conjugates showed the typical purple color, proving that the compounds are in the cationic form. As previously shown, this is mandatory for obtaining cytotoxic activity. The cytotoxicity of the conjugates was determined by SRB assay on a representative selection of human cancer cell lines. For comparison, fibroblasts (murine, NIH 3T3) were used as non-malignant cell lines. The results of these assays are summarized in Table 1 and Figure 2 and Figure 3.

**Figure 4:**
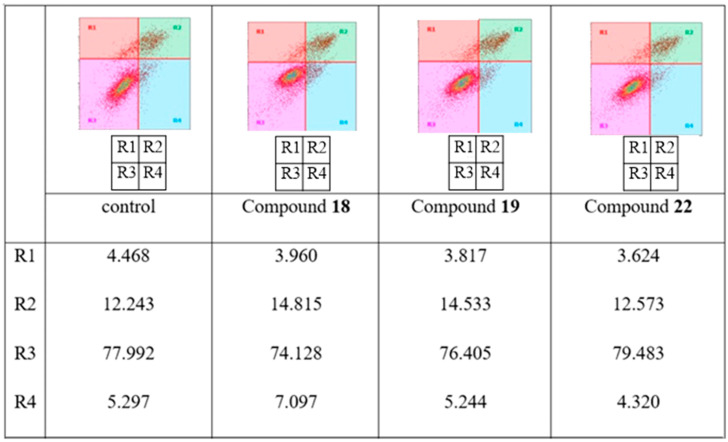
Annexin V-FITC/PI assay: treatment of A375 cells with **18**, **19** and **22** (6.2 μM/4.8 μM/3.0 μM) for 24 h. Examples of density plots determined by flow cytometry (Attune^®^ Cytometric Software v. 1.2.5). R1: necrotic, R2: secondary necrotic/late stage apoptotic, R3: vital, R4: apoptotic.

**Figure 5:**
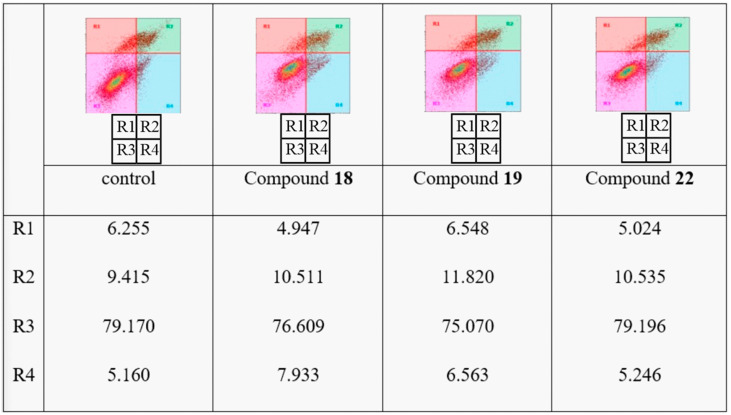
Annexin V-FITC/PI assay: treatment of A375 cells with **18**, **19** and **22** (6.2 μM/4.8 μM/3.0 μM) for 48 h. Examples of density plots determined by flow cytometry (Attune^®^ Cytometric Software v. 1.2.5). R1: necrotic, R2: secondary necrotic/late stage apoptotic, R3: vital, R4: apoptotic.

Evaluation of the results from the Annexin V/FITC/PI assays (Figure 4 and Figure 5) showed compounds **18**, **19** and **22** as acting both via apoptosis and necrosis. An additional investigation of the cell cycle revealed in these compounds and A375 cells, after 1 day of incubation (Figure 4), a decrease in cells in the S and M phase together with an increase in G1/G0. After another 24 h (Figure 5), we observed a further decrease in cells in the S and M phase but an increase in cells in G1/G0 phase. Paralleling prior studies from our group, investigations of mitochondrial function and ATP synthesis from glycolysis and respiration showed these compounds to act as mitocans. Moreover, these experiments revealed a disturbance in cellular energy metabolism as the primary mode of action, thus distinguishing these conjugates from conventional chemotherapeutic drugs. This unique mechanism is responsible for the efficacy of the triterpene–rhodamine conjugates in surmounting resistance often observed for chemotherapeutic agents. Sufficient hydrolytic stability is mandatory for later in vivo applications. Under cell-like conditions, hydrolysis of the conjugates was not observed (a finding that is probably due to the robust amide bonds); however, upon prolonged incubation of the hybrids, partial de-acetylation was observed. However, the rhodamines as well as the parent triterpenoic acids were only of minor cytotoxicity.

From the results of a preliminary structure-activity relationship (SAR) analysis, it can be concluded that the hybrids derived from betulinic acid exhibited greater cytotoxicity than those derived from platanic acid. This finding seems primarily due to the lower solubility (and, as a consequence, a diminished bioavailability) of the latter. Consistent with our previous finding, homopiperazinyl spacered compounds demonstrated superior cytotoxic activity compared to those holding a piperazinyl spacer. The most selective and cytotoxic conjugates are most often those compounds incorporating either a rhodamine B or a rhodamine 101 unit.

## 3. Conclusions

Betulinic acid (**BA**) and platanic acid (**PA**) were used as starting materials to prepare different conjugates with rhodamines. The compounds piperazine and homopiperazine, known from previous studies, were used as linkers between the triterpene and the rhodamine. Two acetyloxy groups were introduced into ring A of the pentacyclic triterpene to potentially increase its cytotoxic activity. The cytotoxicity data (from SRB assays) revealed a significantly higher cytotoxicity for derivatives **13**–**22** derived from betulinic acid, with derivatives **19** (with rhodamine B) and **22** (with rhodamine 101); both of which were provided with a homopiperazinyl spacer; these hybrids showed the best values with EC_50_ = 0.016 and 0.019 μM for A2780 ovarian carcinoma cells. These two compounds also showed the highest selectivity (calculated from the ratio of EC_50_ values) between malignant A2780 cells and non-malignant NIH 3T3 fibroblasts. Cell cycle studies employing A375 melanoma cells revealed that the cells arrested in the G1/G0 phase, and Annexin/FITC/PI staining indicated that these compounds acted in both the apoptosis and necrosis of the tumor cells.

## 4. Experimental Procedure

### 4.1. General

NMR spectra were recorded using the Varian spectrometers (Darmstadt, Germany) DD2 and VNMRS (400 and 500 MHz, respectively). The MS spectra were taken on an Advion expressionL CMS mass spectrometer (Ithaca, NY, USA; positive ion polarity mode, solvent: methanol, solvent flow: 0.2 mL/min, spray voltage: 5.17 kV, source voltage: 77 V, APCI corona discharge: 4.2 μA, capillary temperature: 250 °C, capillary voltage: 180 V, sheath gas: N2). Thin-layer chromatography was performed on pre-coated silica gel plates supplied by Macherey-Nagel (Düren, Germany). IR spectra were recorded on a Spectrum 1000 FT-IR-spectrometer from Perkin Elmer (Rodgau, Germany). The UV/Vis-spectra were recorded on a Lambda 14 spectrometer from Perkin Elmer (Rodgau, Germany); optical rotations were measured at 20 °C using a JASCO-P2000 instrument (JASCO Germany GmbH, Pfungstadt, Germany) The melting points were determined using the Leica hot stage microscope Galen III (Leica Biosystems, Nussloch, Germany). Micro-analyses were performed with an Elementar Vario EL (CHNS) instrument (Elementar Analysensysteme GmbH, Elementar-Straße 1, D-63505, Langenselbold, Germany).

**Figure 6:**
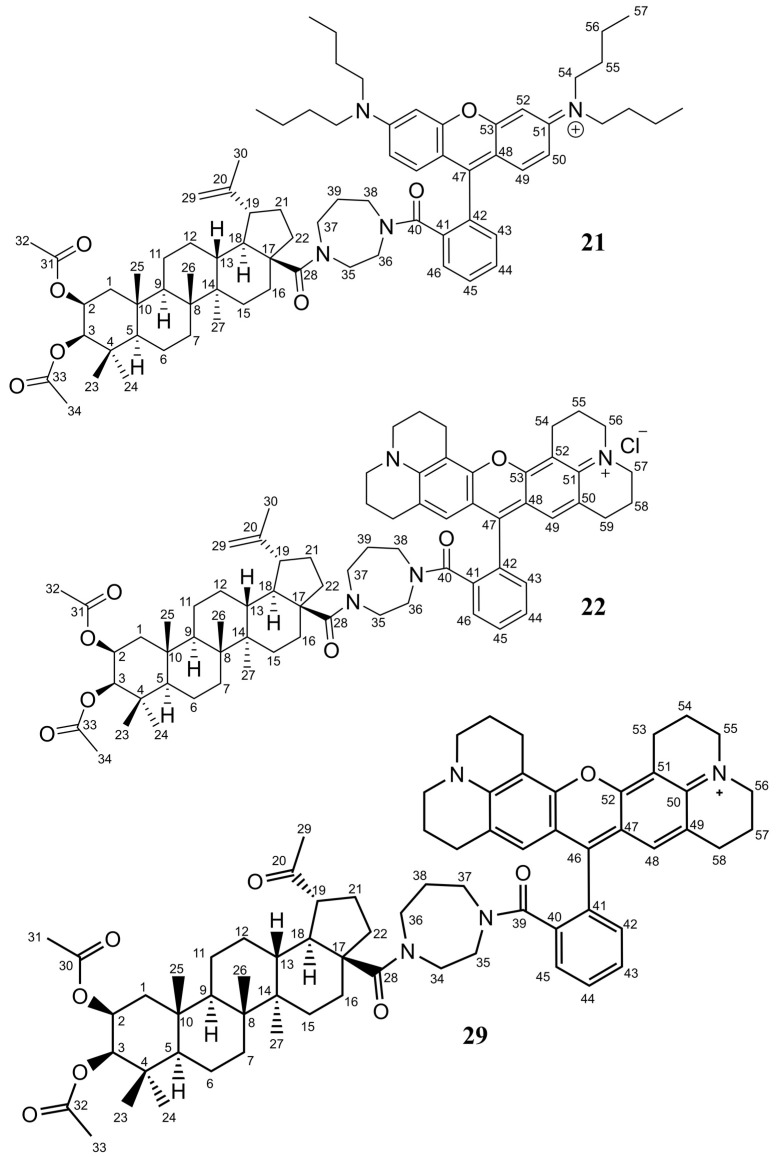
Numbering scheme for hybrids **21**, **22** and **29**.

All compounds were fully characterized by spectroscopy as well as micro-analysis; since all spectroscopic data confirmed the proposed structure, low resolution mass spectrometry was regarded as sufficient for the completion of characterization. High-resolution mass spectrometry does not allow for any conclusion to be drawn about the presence of inorganic impurities. We refrained from measuring optical rotations for the hybrids, due to the deep color of the compounds. The NMR data for compounds **1**–**8** can be found in the literature [52,55,56,57]; the spectra measured for these compounds agreed perfectly with the reported data. A numbering scheme is provided in Figure 6.

Reactions using air- or moisture-sensitive reagents were carried out under an argon atmosphere in dried glassware. All dry solvents were distilled over their respective drying agents, except for DMF, which was distilled and stored under an argon and molecular sieve; Triethylamine was stored over potassium hydroxide. Chemicals and solvents were obtained from local vendors. Betulinic as well as platanic acid were bought from Betulinines (Strbrna Skalice, Czech Republic) and used as received.

Biological assays were performed as previously reported, the cell lines employed were obtained from the Department of Oncologyartin-Luther-University Halle Wittenberg; they were bought from ATCC. For the SRB assay, in short, cells were seeded into 96-well plates on day zero at appropriate cell densities to prevent confluence of the cells during the period of the experiment. After 24 h, the cells were treated with different concentrations, but the final concentration of DMSO/DMF never exceeded 0.5%, which was non-toxic to the cells. After 72 h of treatment, the supernatant from the 96-well plates was discarded, then the cells were fixed with 10% trichloroacetic acid and allowed to rest at 4 °C. After 24 h of fixation, the cells were washed in a strip washer and then dyed with SRB solution (200 μL, 10 mM) for 20 min. The plates were washed four times with 1% acetic acid to remove the excess of the dye and allowed to air-dry overnight. Tris base solution (200 μL, 10 mM) was added to each well. The absorbance was measured with a 96-well plate reader from Tecan Spectra (Tecan Germany GmbH, Crailsheim, Germany).

### 4.2. Syntheses

#### 4.2.1. General Procedure for Acetylations (GPA)

Acetylations were performed in dry DCM (100 mL) with acetic anhydride in the presence of NEt_3_ and DMAP (catal.) for 24 h as previously described, followed by the usual aq. work-up and chromatography of the crude product to yield the corresponding acetates. 

#### 4.2.2. General Procedure for the Synthesis of (Homo)Piperazinyl Amides (GPB)

A solution of the corresponding carboxylic acid (0.9 mmol) in dry DCM (25 mL) was treated with oxalyl chloride (0.3 mL, 3.6 mmol)/DMF (catal.) for 30 min, followed by the evaporation of the volatiles; the residue was dissolved in dry DCM (20 mL) and allowed to react with a solution of (homo)piperazine (1.8 mmol) in dry DCM (8 mL)/DMAP (catal.) for 24 h. The usual work-up, followed by chromatography, furnished the (homo)piperazinyl amides.

#### 4.2.3. General Procedure for the Synthesis of the Rhodamine Conjugates (GPC)

Reaction of the rhodamine (1.0 eq, 0.88 mmol) in dry DCM (25 mL) with oxalyl chloride (5 eq., 0.7 mL, 4.4 mmol) and DMAP (catal.) for 30 min, followed by adding a solution of the triterpenoic (homo)piperazinyl amide (1.0 eq., 0.8 mmol) in dry DCM (10 mL) in the presence of DMAP (catal.) and NEt_3_ (1.5 eq., 0.4 mL, 1.2 mmol), followed by stirring for 1 d at 21 °C, the usual work-up and chromatography gave the corresponding rhodamine conjugates. 

#### 4.2.4. 3-Oxolup-20(29)-en-28-oic Acid (Betulonic Acid) (**1**) 

A silica-supported Jones oxidation [Jones reagent freshly prepared from CrO_3_ (2.5 g, 25.1 mmol), H_2_SO_4_ (2.5 mL) and H_2_O (10 mL)] of **BA** (10.0 g, 21.9 mmol) in acetone (500 mL), followed by the usual work-up and Soxhlet extraction with ether (600 mL, 6 h) and chromatography (SiO_2_, hexanes/ethyl acetate, 8:2) gave **1** (6.23 g, 76%) as a colorless solid [23,33]; R_f_ = 0.42 (SiO_2_, hexanes/ethyl acetate, 7:1); m.p. = 246 °C (decomp.) (lit.: 249–250 °C); ${\left[\alpha \right]}_{D}^{20}$ = +37.4° (*c* 0.283, CHCl_3_) (lit.: ${\left[\alpha \right]}_{D}^{20}$ = +33.8° (*c* 0.33, CHCl_3_); MS (ESI, MeOH): *m*/*z* = 453.3 (36%, [M-H]^−^), 907.2 (100%, [2M-H]^−^), 930.1 (72%, [2M-2H+Na]^−^).

#### 4.2.5. 3,20-Dioxo-30-Norlupan-28-oic Acid (**2**)

A silica-supported Jones oxidation of **PA** (10.0 g, 21.0 mmol), as described above, followed by chromatography (SiO_2_, hexanes/ethyl acetate, 8:2), gave **2** (7.3 g, 82%) as a colorless solid [55]; R_f_ = 0.64 (SiO_2_, hexanes/ethyl acetate, 1:1); m.p. = 230–232 °C (lit.: 230 °C); ${\left[\alpha \right]}_{D}^{20}$ = +5.0° (*c* 0.05, CHCl_3_); (lit.: ${\left[\alpha \right]}_{D}^{20}$ = +4.2° (*c* 0.308, CHCl_3_); MS (ESI, MeOH): *m/z* 455.7 (54%, [M-H]^−^), 911.5 (48%, [2M-H]^−^), 934.4 (100%, [2M-2H+Na]^−^).

#### 4.2.6. 2-Hydroxy-3-oxolupa-1,20(29)-dien-28-oic Acid (**3**) 

A solution of **1** (8.55 g, 18.8 mmol) in *tert*-butanol (500 mL) and dry THF (70 mL) was allowed to react with potassium *t*-butanolate (30.0 g, 0.267 mol) for 3 h at 50 °C with dry air continuously bubbling through the reaction mixture; the volatiles were removed under diminished pressure, and the residue was dissolved in ethyl acetate; washing with aq. HCl (5%, 20 mL) and the extraction of the aq. phase with DCM (3 × 10 mL), followed by a removal of the solvents and chromatography (SiO_2_, hexanes/ethyl acetate: ethyl acetate: 5% → 20%) gave **3** (7.41 g, 97%) as a colorless solid [56,57]; R_f_ = 0.68 (SiO_2_, hexanes/ethyl acetate, 3:1); m.p. = 200–203 °C (lit.: 203–205 °C); ${\left[\alpha \right]}_{D}^{20}$= +11.7° (*c* 0.5, CHCl_3_) (lit.: ${\left[\alpha \right]}_{D}^{20}$ = 12.0° (*c* 0.56, CHCl_3_); MS (ESI, MeOH): *m*/*z* = 467.2 (76%, [M-H]^−^), 935.4 (100%, [2M-H]^−^), 958.1 (86%, [2M-2H+Na]^−^).

#### 4.2.7. 2-Hydroxy-3,20-dioxo-30-norlup-1-en-28-oic Acid (**4**) 

Reaction of **2** (7.3 g, 16 mmol) with *tert*-butanol (400 mL)/THF (80 mL) and potassium-*tert*-butanolate (25.8 g, 0.23 mol, 14 eq.)/air for 2 at 50 °C, as described above, gave **4** (4.88 g, 75%) as a viscous oil that was used without further purification; an analytical sample showed m.p. = 224–227 °C (lit.: [56] m.p. = 224–228 °C); ${\left[\alpha \right]}_{D}^{20}$ = +9.0° (c 0.56, MeOH) (lit.: [56] ${\left[\alpha \right]}_{D}^{20}$ = +9.2° (*c* 0.71, MeOH); MS (ESI, MeOH): *m*/*z* 472.3 (14%, [M+H]^+^), 493.1 (100%, [M+Na]^+^), 509.1 (32%, [M+K]^+^).

#### 4.2.8. 2β,3β-Dihydroxylup-20(29)-en-28-oic Acid (**5**) 

To a solution of **3** (8.53 g, 18.2 mmol) in THF (200 mL) and methanol (40 mL), NaBH_4_ (1.0 g, 26.4 mmol) was added in several portions, and the mixture was stirred at 21 °C for 2 days. The usual aq. work-up, followed by chromatography (SiO_2_, hexanes/ethyl acetate, ethyl acetate: 10% → 30%) gave **5** (5.79 g, 79%) as a colorless solid; R_f_ = 0.54 (SiO_2_, hexanes/ethyl acetate, 1:1); m.p. = 270–273 °C (decomp) (lit.: [55] 273–276 °C); ${\left[\alpha \right]}_{D}^{20}$ = +32.4° (*c* 0.212, CHCl_3_) (lit.: ${\left[\alpha \right]}_{D}^{20}$ = +31.1° (*c* 0.262, pyridine); MS (ESI, MeOH): *m*/*z* = 473.3 (18%, [M+H]^+^), 490.4 (10%, [M+NH_4_]^+^), 495.8 (100%, [M+Na]^+^).

#### 4.2.9. 2β,3β-Dihydroxy-20-oxo-30-norlupan-28-acid (**6**)

Reduction of **4** (4.9 g, 10.4 mmol) with NaBH_4_ (1.6 g, 42 mmol) in THF (100 mL) and MeOH (20 mL) for 1 day at 21 °C followed by chromatography (SiO_2_, hexanes/ethyl acetate, 1:1) gave **6** (4.3 g, 87%) as a colorless solid; R_f_ = 0.31 (SiO_2_, hexanes/ethyl acetate, 1:1); m.p. = 264–268 °C (lit.: [55] 265–269 °C); ${\left[\alpha \right]}_{D}^{20}$ = +10.7° (*c* 0.25, CHCl_3_) (lit.: [55] ${\left[\alpha \right]}_{D}^{20}$ = +11.2° (*c* 0.130, CHCl_3_); MS (ESI, MeOH): *m*/*z* 473.1 (52%, [M-H]^−^), 947.3 (100%, [2M-H] ^−^).

#### 4.2.10. 2β,3β-Bis(acetyloxy)-lup-20(29)-en-28-oic Acid (**7**) 

According to the GPA from **5** (8.53 g, 18.2 mmol), followed by chromatography (SiO_2_, hexanes/ethyl acetate, 8:2), **7** (8.6 g, 86%) was obtained as a colorless solid; R_f_ = 0.63 (SiO_2_, hexanes/ethyl acetate, 7:1); m.p. = 261–264 °C (lit.: [55] 260–265 °C); ${\left[\alpha \right]}_{D}^{20}$= +33.8° (*c* 0.2, CHCl_3_) (lit.: [55] ${\left[\alpha \right]}_{D}^{20}$= +34.6° (*c* 0.199, CHCl_3_); MS (ESI, MeOH): *m*/*z* = 437.1 (8%, [M+H-2HOAc]^+^), 497.5 (10%, [M+H-HOAc]^+^), 1135.1 (100%, [2M+Na]^+^).

#### 4.2.11. 2β,3β-2,3-Bis(acetyloxy)-20-oxo-30-norlupan-28-oic Acid (**8**) 

According to the GPA from **6** (4.3 g, 9.5 mmol), followed by chromatography (SiO_2_, hexanes/ethyl acetate, 8:2), **8** (4.9 g, 92%) was obtained as a colorless solid; R_f_ = 0.33 (SiO_2_, hexanes/ethyl acetate, 3:1); m.p. = 148–150 °C (lit.: 150 °C); ${\left[\alpha \right]}_{D}^{20}$ = +6.7° (*c* 0.11, CHCl_3_) (lit.: ${\left[\alpha \right]}_{D}^{20}$ = +6.9° (*c* 0.146, CHCl_3_); MS (ESI, MeOH): *m*/*z* 439.2 (28%, [M+H-HOAc]^+^), 499.3 (32%, [M+H-2HOAc]^+^), 576.4 (100%, [M+NH_4_]^+^), 581.1 (48%, [M+Na]^+^).

#### 4.2.12. 2β,3β-Bis(acetyloxy)-28-(1-piperazinyl)lup-20(29)en-28-one (**9**)

According to the GPB from **7** (1.0 g, 2.0 mmol), followed by chromatography (SiO_2_, CHCl_3_/MeOH, 9:1), **9** (744 mg, 70%) was obtained as a colorless solid; R_f_ = 0.55 (SiO_2_, CHCl_3_/MeOH, 9:1); m.p. = 192 °C; ${\left[\alpha \right]}_{D}^{20}$ = +14.95° (*c* 0.137, CHCl_3_); IR (ATR): ν = 2940*m*, 1740*vs*, 1624*w*, 1365*s*, 1245*vs*, 1189*s*, 1031*m* cm^−1^; ^1^H NMR (500 MHz, CDCl_3_): δ = 5.30 (*dd*, *J* = 7.1, 3.8 Hz, 1H, 2-H), 4.73–4.70 (*m*, 1H, 29-H_a_), 4.60–4.57 (*m*, 2H, 29-H_b_, 3-H), 2.98–2.89 (*m*, 9H, 19-H, 35-H, 36-H, 37-H, 38-H), 2.84 (*td*, *J* = 13.1, 3.4 Hz, 1H, 13-H), 2.08–2.05 (*m*, 1H, 16-H_a_), 2.02 (*s*, 3H, 34-H), 2.01 (*s*, 3H, 32-H), 1.99–1.75 (*m*, 3H, 1-H_a_, 22-H_a_, 21-H_a_), 1.74–1.64 (*m*, 4H, 12-H_a_, 30-H), 1.60–1.44 (*m*, 4H, 6-H, 16-H_b_, 18-H), 1.45–1.28 (*m*, 7H, 11-H, 21-H_b_, 7-H, 22-H_b_, 15-H_a_), 1.30–1.19 (*m*, 3H, 1-H_b_, 9-H, 15-H_b_), 1.10 (*s*, 3H, 26-H), 1.01 (*s*, 3H, 25-H), 0.94 (*s*, 6H, 27-H, 24-H), 0.93–0.91 (*m*, 2H, 5-H, 12-H_b_), 0.87 (*s*, 3H, 23-H) ppm; ^13^C NMR (125 MHz, CDCl_3_): δ = 173.7 (C-28), 170.9 (C-33), 170.4 (C-31), 151.1 (C-20), 109.5 (C-29), 78.1 (C-3), 69.8 (C-2), 55.5 (C-5), 54.7 (C-17), 52.8 (C-18), 51.4 (C-9), 45.8 (C-19), 45.5 (C-35, C-36, C-37, C-38), 42.4 (C-14), 42.2 (C-1), 40.9 (C-8), 37.5 (C-4), 37.2 (C-10), 37.0 (C-13), 36.0 (C-22), 34.4 (C-7), 32.7 (C-16), 31.4 (C-21), 29.9 (C-15), 29.1 (C-23), 25.7 (C-12), 21.4 (C-11), 21.4 (C-34), 21.0 (C-32), 19.8 (C-30), 18.2 (C-6), 17.6 (C-25), 16.9 (C-26), 16.4 (C-24), 14.7 (C-27) ppm; MS (ESI, MeOH/CHCl_3_): *m*/*z* 626.6 (100%, [M+H]^+^), 648.1 (35%, [M+Na]^+^); analysis calcd for C_38_H_60_N_2_O_5_ (624.91): C 73.04, H 9.68, N 4.48; found: C 72.83, H 9.85; N 4.17.

#### 4.2.13. 2β,3β-Bis(acetyloxy)-28-(1-hexahydro-1H-1,4-diazepin-1-yl)lup-20(29)en-28-one (**10**)

According to the GPB from **7** (1.0 g, 2.0 mmol), followed by chromatography (SiO_2_, CHCl_3_/MeOH, 9:1), **10** (1.1 g, 85%) was obtained as a colorless solid; R_f_ = 0.5 (SiO_2_, CHCl_3_/MeOH, 9:1); m.p. = 149–153 °C; ${\left[\alpha \right]}_{D}^{20}$ = +10.89° (*c* 0.064, CHCl_3_); IR (ATR): ν = 2941*m*, 1741*s*, 1625*w*, 1367*s*, 1231*vs*, 1188*s*, 1031*m* cm^−1^; ^1^H NMR (400 MHz, CDCl_3_): δ = 5.29–5.29 (*m*, 1H, 2-H), 4.73–4.69 (*m*, 1H, 29-H_a_), 4.60–4.56 (*m*, 2H, 29-H_b_, 3-H), 3.79–3.58 (*m*, 10H, 35-H, 36-H, 37-H, 38-H, 39-H), 3.00–2.83 (*m*, 2H, 19-H, 13-H), 2.15–2.08 (*m*, 1H, 16-H_a_), 2.02 (*s*, 3H, 34-H), 2.01 (*s*, 4H, 32-H, 1-H_a_), 1.98–1.94 (*m*, 1H, 22-H_a_), 1.87–1.77 (*m*, 1H, 21-H_a_), 1.75–1.62 (*m*, 1H, 12-H_a_), 1.67 (*s*, 3H, 30-H), 1.60–1.44 (*m*, 4H, 16-H_b_, 18-H, 6-H), 1.44–1.19 (*m*, 9H, 11-H, 15-H_a_, 21-H_b_, 7-H, 22-H_b_, 1-H_b_, 9-H), 1.19–1.03 (*m*, 1H, 15-H), 1.09 (*s*, 3H, 26-H), 1.01 (*s*, 3H, 25-H), 0.95 (*s*, 3H, 24-H), 0.94 (*s*, 3H, 27-H), 0.94–0.91 (*m*, 2H, 12-H_b_, 5-H), 0.87 (*s*, 3H, 23-H) ppm; ^13^C NMR (125 MHz, CDCl_3_): δ = 174.8 (C-28), 170.9 (C-33), 170.4 (C-31), 151.1 (C-20), 109.5 (C-29), 78.1 (C-3), 69.8 (C-2), 55.5 (C-5), 55.1 (C-17), 53.0 (C-18), 51.5 (C-9), 47.0 (C-35, C-36, C-37, C-38, C-39), 45.8 (C-19), 42.4 (C-14), 42.3 (C-1), 41.0 (C-8), 37.5 (C-4), 37.2 (C-10), 37.0 (C-13), 36.3 (C-22), 34.4 (C-7), 32.4 (C-16), 31.5 (C-21), 29.9 (C-15), 29.1 (C-23), 25.7 (C-12), 21.4 (C-11), 21.4 (C-34), 21.0 (C-32), 19.9 (C-30), 18.2 (C-6), 17.6 (C-25), 16.9 (C-26), 16.4 (C-24), 14.8 (C-27) ppm; MS (ESI, MeOH/CHCl_3_): *m*/z 640.2 (100%, [M+H]^+^); analysis calcd for C_39_H_62_N_2_O_5_ (638.93): C 73.31, H 9.78, N 4.38; found: C 73.12, H 9.97; N 4.19.

#### 4.2.14. 2β,3β-Bis(acetyloxy)-28-(1-piperazinyl)-30-norlupane-20,28-dione (**11**) 

According to the GPB from **8** (1.0 g, 1.8 mmol), followed by chromatography (SiO_2_, CHCl_3_/MeOH, 9:1), **11** (1.25 g, 90%) was obtained as a colorless solid; R_f_ = 0.4 (SiO_2_, CHCl_3_/MeOH, 9:1); m.p. = 175–179 °C; ${\left[\alpha \right]}_{D}^{20}$ = −0.75° (*c* 0.062, CHCl_3_); IR (ATR): ν = 2942*m*, 1740*vs*, 1629*w*, 1366*s*, 1245*vs*, 1192*s*, 1030*s* cm^−1^; ^1^H NMR (500 MHz, CDCl_3_): δ = 5.33–5.28 (*m*, 1H, 2-H), 4.59 (*d*, *J* = 3.9 Hz, 1H, 3-H), 3.30–3.16 (*m*, 1H, 19-H), 3.05–2.95 (*m*, 8H, 34-H, 35-H, 36-H, 37-H), 2.64 (*td*, *J* = 12.5, 3.9 Hz, 1H, 13-H), 2.16 (*s*, 3H, 29-H), 2.02 (*s*, 3H, 31-H), 2.01 (*s*, 3H, 33-H), 2.13–1.83 (*m*, 5H, 18-H, 16-H_a_,1-H_a_, 22-H_a_, 21-H_a_), 1.69–1.12 (*m*, 12H, 16-H_b_, 6-H, 22-H_b_, 21-H_b_, 7-H, 11-H, 15-H_a_, 1-H_b,_ 15-H_b_), 1.09 (*s*, 3H, 25-H), 1.01 (*s*, 3H, 24-H), 1.07–0.94 (*m*, 2H, 12-H), 0.97 (*s*, 3H, 27-H), 0.92 (*s*, 3H, 26-H), 0.94–0.89 (*m*, 1H, 5-H), 0.87 (*s*, 3H, 27-H) ppm; ^13^C NMR (101 MHz, CDCl_3_): δ = 212.7 (C-20), 173.7 (C-28), 170.8 (C-32), 170.3 (C-30), 78.0 (C-3), 69.8 (C-2), 55.5 (C-5), 54.6 (C-17), 52.5 (C-18), 51.3 (C-9), 50.0 (C-19), 45.0 (C-35, C-36, C-38, C-39), 42.3 (C-14), 42.1 (C-1), 40.8 (C-8), 37.6 (C-4), 37.2 (C-10), 36.0 (C-13), 35.7 (C-22), 34.2 (C-7), 32.2 (C-16), 30.5 (C-29), 29.9 (C-15), 29.1 (C-23), 28.8 (C-21), 27.5 (C-12), 21.4 (C-11), 21.4 (C-33), 21.0 (C-31), 18.1 (C-6), 17.6 (C-24), 16.9 (C-25), 16.3 (C-26), 14.7 (C-27) ppm; MS (ESI, MeOH): *m*/*z* 627.7 (100%, [M+H]^+^), 1254.1 (20%, [2M+H]^+^); analysis calcd for C_37_H_58_N_2_O_6_ (626.88): C 70.89, H 9.33, N 4.47; found: C 70.63, H 9.54; N 4.19.

#### 4.2.15. 2β,3β-Bis(acetyloxy)-28-(hexahydro-1H-1,4-diazepin-1-yl)-30-norlupane-20,28-dione (**12**)

According to the GPB from **8** (1.0 g, 1.8 mmol), followed by chromatography (SiO_2_, CHCl_3_/MeOH, 9:1), **12** (0.8 g, 70%) was obtained as a colorless solid; R_f_ = 0.2 (SiO_2_, CHCl_3_/MeOH, 9:1); m.p. = 185–189 °C; ${\left[\alpha \right]}_{D}^{20}$ = −13.09° (c 0.067, CHCl_3_); IR (ATR): ν = 2940*m*, 1740*vs*, 1624*w*, 1365*s*, 1245*vs*, 1189*s*, 1031*m* cm^−1^; ^1^H NMR (500 MHz, CDCl_3_): δ = 5.32–5.28 (*m*, 1H, 2-H), 4.65–4.52 (*m*, 1H, 3-H), 3.99–3.43 (*m*, 8H, 34-H, 35-H, 36-H, 37-H), 3.33–3.14 (*m*, 1H, 19-H), 2.65 (*td*, *J* = 12.9, 12.4, 4.1 Hz, 1H, 13-H), 2.17 (*s*, 3H, 29-H), 2.21–2.03 (*m*, 2H, 16-H_a_, 18-H), 2.02 (*s*, 3H, 31-H), 2.01 (*s*, 3H, 33-H), 2.04–1.77 (*m*, 3H, 1-H_a_, 22-H_a_, 21-H_a_), 1.69–1.31 (*m*, 9H, 16-H_b_, 6-H, 22-H_b_, 21-H_b_, 7-H, 11-H), 1.31–1.10 (*m*, 4H, 15-H, 9-H, 1-H_b_), 1.09 (*s*, 3H, 25-H), 1.10–0.95 (*m*, 2H, 12-H), 1.01 (*s*, 3H, 24-H), 0.97 (*s*, 3H, 27-H), 0.93 (*s*, 3H, 26-H), 0.95–0.88 (*m*, 1H), 0.87 (*s*, 3H, 23-H) ppm; ^13^C NMR (101 MHz, CDCl_3_): δ = 212.7 (C-20), 175.0 (C-28), 170.8 (C-32), 170.3 (C-30), 78.0 (C-3), 69.8 (C-2), 55.5 (C-5), 55.0 (C-17), 52.6 (C-18), 51.3 (C-9), 50.0 (C-19), 46.8 (C-34, C-35, C-36, C-37), 42.3 (C-14), 42.1 (C-1), 40.9 (C-8), 37.5 (C-4), 37.4 (C-10), 36.0 (C-13), 35.9 (C-22), 34.2 (C-7), 31.8 (C-16), 30.5 (C-29), 30.0 (C-15), 29.1 (C-23), 28.9 (C-21), 27.4 (C-12), 21.4 (C-11), 21.4 (C-33), 21.0 (C-31), 18.1 (C-6), 17.6 (C-24), 16.9 (C-25), 16.3 (C-26), 14.8 (C-27) ppm; MS (ESI, MeOH/CHCl_3_): *m*/*z* 642.7 (100%, [M+H]^+^), 1282 (20%, [2M+H]^+^); analysis calcd for C_38_H_60_N_2_O_6_ (640.90): C 71.21, H 9.44, N 4.37; found: C 70.87, H 9.62; N 4.17.

#### 4.2.16. 9-[2-[[4-(2β,3β-Bis(acetyloxy)-lup-20(29)-en-28-oyl)-1-piperazinyl]carbonyl]phenyl]-3,6-bis(dimethylamino)-xanthylium Chloride (**13**) 

According to the GPC from **9** (200 mg, 0.32 mmol), followed by chromatography (SiO_2_, CHCl_3_/MeOH, 9:1), **13** (233 mg, 71%) was obtained as a violet solid; R_f_ = 0.54 (SiO_2_, CHCl_3_/MeOH, 9:1); m.p. = 270 °C (decomp.); UV-Vis (MeOH): λ_max_ (log ε) = 556 nm (4.97); IR (ATR): ν = 3395*br*, 2936*w*, 1738*w*, 1632*m*, 1592*s*, 1534*w*, 1493*m*, 1407*m*, 1365*m*, 1343*s*, 1284*w*, 1258*m*, 1232*m*, 1185*s*, 1134*m*, 1056*w*, 1031*m*, 1003*m*, 982*w*, 925*m*, 880*w*, 821*w*, 786*m*, 758*w*, 699*m*, 661*w*, 602*w*, 580*w*, 516*m*, 490*w*, 458*w* cm^−1^; ^1^H NMR (500 MHz, CDCl_3_): δ = 7.71–7.65 (*m*, 2H, 42-H, 44-H), 7.56–7.50 (*m*, 1H, 45-H), 7.40–7.33 (*m*, 1H, 43-H), 7.29–7.18 (*m*, 1H, 49-H), 7.12–6.98 (*m*, 1H, 48-H), 6.90–6.80 (*m*, 1H, 51-H), 5.31–5.26 (*m*, 1H, 2-H), 4.69–4.64 (*m*, 1H, 29-H_a_), 4.59–4.52 (*m*, 2H, 29-H_b_, 3-H), 3.34 (*s*, 6H, 53-H), 2.90–2.82 (*m*, 1H, 19-H), 2.78–2.68 (*m*, 1H, 13-H), 2.00 (*s*, 3H, 32-H), 1.99 (*s*, 3H, 34-H), 1.98–1.95 (*m*, 1H, 16-H_a_), 1.95–1.92 (*m*, 1H, 1-H_a_), 1.84–1.77 (*m*, 1H, 12-H_a_), 1.75–1.57 (*m*, 5H, 21-H_a_, 12-H_a_, 30-H), 1.56–1.41 (*m*, 4H, 6-H, 16-H_b_, 18-H), 1.40–1.17 (*m*, 10H, 22-H_b_, 7-H, 21-H, 11-H, 15-H_a_, 1-H_b_, 9-H), 1.06 (*s*, 3H, 26-H), 0.99 (*s*, 3H, 25-H), 0.89 (*s*, 5H, 27-H, 12-H_b_, 5-H), 0.87 (*s*, 3H, 24-H), 0.85 (*s*, 3H, 23-H) ppm; ^13^C NMR (125 MHz, CDCl_3_): δ = 174.3 (C-28), 170.8 (C-33), 170.3 (C-31), 167.7 (C-39), 157.6 (C-50), 157.5 (C-52), 157.5 (C-46), 151.0 (C-20), 135.2 (C-41), 132.1 (C-49), 130.6 (C-40), 130.4 (C-42, C-44), 130.2 (C-43), 127.7 (C-45), 114.6 (C-48), 114.0 (C-47), 109.5 (C-29), 96.9 (C-51), 78.1 (C-3), 69.7 (C-2), 55.4 (C-5), 54.7 (C-17), 52.6 (C-18), 51.3 (C-9), 45.8 (C-19), 42.3 (C-1), 42.1 (C-14), 41.3 (C-53), 40.9 (C-8), 37.5 (C-4), 37.1 (C-10), 36.9 (C-13), 35.9 (C-22), 34.3 (C-7), 32.6 (C-16), 31.3 (C-21), 29.8 (C-15), 29.0 (C-23), 25.5 (C-12), 21.3 (C-34), 21.3 (C-11), 20.9 (C-32), 19.6 (C-30), 18.1 (C-6), 17.6 (C-25), 16.9 (C-26), 16.3 (C-24), 14.6 (C-27) ppm; MS (ESI, MeOH/CHCl_3_): *m*/*z* 993.5 (100%, [M-Cl]^+^); analysis calcd for C_62_H_81_N_4_O_7_Cl (1029.80): C 72.31, H 7.93, N 5.44; found: C 72.05, H 8.06; N 5.20.

#### 4.2.17. 9-[2-[[4-(2β,3β-Diacetoxy-lup-20(29)-en-28-oyl)-1-piperazinyl]carbonyl]phenyl]-3,6-bis(diethylamino)-xanthylium Chloride (**14**)

According to the GPC from **9** (260 mg, 0.42 mmol), followed by chromatography (SiO_2_, CHCl_3_/MeOH, 9:1), **14** (320 mg, 71%) was obtained as a violet solid; R_f_ = 0.53 (SiO_2_, CHCl_3_/MeOH, 9:1); m.p. = 265 °C (decomp.); UV-Vis (MeOH): λ_max_ (log ε) = 560 nm (4.97); IR (ATR): ν = 2937*br*, 1737*w*, 1631*m*, 1586*vs*, 1528*w*, 1506*w*, 1466*m*, 1411*s*, 1335*s*, 1300*w*, 1272*w*, 1245*s*, 1179*vs*, 1131*m*, 1072*m*, 1031*w*, 1003*m*, 978*m*, 921*m*, 870*w*, 823*m*, 787*w*, 757*w*, 683*m*, 665*w*, 579*w*, 544*w*, 496*w*, 440*w* cm^−1^; ^1^H-NMR (400 MHz, CDCl_3_): δ = 7.71–7.62 (*m*, 2H, 42-H, 43-H), 7.55–7.49 (*m*, 1H, 45-H), 7.38–7.32 (*m*, 1H, 44-H), 7.32–7.21 (*m*, 1H, 49-H), 7.05–6.92 (*m*, 1H, 47-H), 6.85–6.77 (*m*, 1H, 51-H), 5.30–5.26 (*m*, 1H, 2-H), 4.68–4.65 (*m*, 1H, 29-H_a_), 4.57 (*d*, *J* = 3.8 Hz, 1H, 3-H), 4.56–4.53 (*m*, 1H, 29-H_b_), 3.73–3.23 (*m*, 10H, 53-H, 35-H, 36-H, 37-H, 38-H), 2.88 (*dt*, *J* = 11.2, 6.1 Hz, 1H, 19-H), 2.78–2.68 (*m*, 1H, 13-H), 2.00 (*s*, 3H, 32-H), 1.99 (*s*, 3H, 34-H), 2.05–1.90 (*m*, 2H, 16-H_a_, 1-H_a_), 1.86–1.78 (*m*, 1H, 22-H_a_), 1.75–1.56 (*m*, 2H, 21-H_a_, 12-H_a_), 1.63 (*s*, 3H, 30-H), 1.56–1.43 (*m*, 4H, 6-H, 16-H_b_, 18-H), 1.42–1.17 (*m*, 12H, 7-H, 22-H_b_, 21-H_b_, 11-H, 54-H, 1-H_b_, 9-H, 15-H_a_), 1.17–1.02 (*m*, 1H, 15-H_b_, 12-H_b_), 1.06 (*s*, 3H, 26-H), 0.99 (*s*, 3H, 25-H), 0.90 (*s*, 4H, 27-H, 5-H), 0.88 (*s*, 3H, 24-H), 0.86 (*s*, 3H, 23-H) ppm; ^13^C-NMR (100 MHz, CDCl_3_): δ = 174.3 (C-28), 170.8 (C-33), 170.4 (C-31), 167.8 (C-39), 157.9 (C-52), 155.8 (C-50), 155.7 (C-46), 151.0 (C-20), 135.1 (C-41), 132.4 (C-49), 130.8 (C-40), 130.5 (C-44), 130.4 (C-42), 130.3 (C-43), 127.7 (C-45), 114.5 (C-48), 114.0 (C-47), 109.6 (C-29), 96.6 (C-51), 78.1 (C-3), 69.7 (C-2), 55.4 (C-5), 54.8 (C-17), 52.6 (C-18), 51.3 (C-9), 46.4 (C-35, C-36, C-37, C-38), 46.4 (C-53), 45.8 (C-19), 42.3 (C-14), 42.2 (C-1), 40.9 (C-8), 37.5 (C-4), 37.1 (C-10), 37.0 (C-13), 36.0 (C-22), 34.3 (C-7), 32.6 (C-16), 31.3 (C-21), 29.8 (C-15), 29.1 (C-23), 25.6 (C-12), 21.4 (C-34), 21.3 (C-11), 21.0 (C-32), 19.6 (C-30), 18.1 (C-6), 17.6 (C-25), 16.9 (C-26), 16.3 (C-24), 14.6 (C-27), 12.8 (C-54) ppm; MS (ESI, MeOH): *m*/*z* 1050.1 (100%, [M-Cl]^+^); analysis calcd for C_66_H_89_N_4_O_7_Cl (1085.67): C 75.46, H 8.54, N 5.33; found: C 75.17, H 8.86, N 5.20.

#### 4.2.18. 9-[2-[[4-(2β,3β-Diacetoxy-lup-20(29)-en-28-oyl)-1-piperazinyl]carbonyl]phenyl]-3,6-bis(dipropylamino)-xanthylium Chloride (**15**)

According to the GPC from **9** (120 mg, 0.19 mmol), followed by chromatography (SiO_2_, CHCl_3_/MeOH, 9:1), **15** (95 mg, 43%) was obtained as a purple solid; R_f_ = 0.3 (SiO_2_, EtOAc/MeOH, 9:1); m.p. = 228 °C; UV-Vis (MeOH): λ_max_ (log ε) = 564 nm (5.02); IR (ATR): ν = 3373*br*, 2936*w*, 1737*w*, 1631*m*, 1587*vs*, 1467*m*, 1411*m*, 1337*s*, 1300*w*, 1252*m*, 1230*s*, 1177*vs*, 1132*m*, 1100*m*, 1031*w*, 1002*w*, 940*m*, 877*w*, 823*w*, 758*w*, 706*w*, 665*w*, 600*w*, 575*w*, 508*w* cm^−1^; ^1^H NMR (500 MHz, CDCl_3_): δ = 7.67–7.61 (*m*, 2H, 42-H, 43-H), 7.55–7.46 (*m*, 1H, 45-H), 7.36–7.29 (*m*, 1H, 44-H), 7.29–7.20 (*m*, 1H, 49-H), 7.07–6.86 (*m*, 1H, 48-H), 6.77–6.70 (*m*, 1H, 51-H), 5.30–5.24 (*m*, 1H, 2-H), 4.70–4.62 (*m*, 1H, 29-H_a_), 4.58–4.52 (*m*, 2H, 29-H_b_, 3-H), 3.56–3.23 (*m*, 10H, 35-H, 36-H, 37-H, 38-H, 53-H), 2.86 (*td*, *J* = 11.2, 5.0 Hz, 2H, 19-H), 2.72 (*td*, *J* = 12.8, 3.2 Hz, 1H, 13-H), 1.99 (*s*, 3H, 32-H), 1.98 (*s*, 3H, 34-H), 2.04–1.87 (*m*, 2H, 16-H_a_, 1-H_a_), 1.84–1.76 (*m*, 1H, 22-H_a_), 1.76–1.65 (*m*, 4H, 54-H, 21-H_a_, 12-H_a_), 1.62 (*s*, 3H, 30-H), 1.56–1.38 (*m*, 4H, 6-H, 16-H_b_, 18-H), 1.39–1.17 (*m*, 9H, 22-H_b_, 7-H, 11-H, 21-H_b_, 15-H_a_, 1-H_b_, 9-H), 1.12–1.01 (*m*, 1H, 15-H_b_), 1.05 (*s*, 3H, 26-H), 1.01–0.95 (*m*, 6H, 55-H, 25-H), 0.94–0.87 (*m*, 2H, 12-H_b_, 5-H), 0.89 (*s*, 3H, 27-H), 0.87 (*s*, 3H, 24-H), 0.84 (*s*, 3H, 23-H) ppm; ^13^C NMR (125 MHz, CDCl_3_): δ = 174.1 (C-28), 170.7 (C-33), 170.2 (C-31), 167.7 (C-39), 157.7 (C-52), 156.1 (C-50), 156.1 (C-46), 150.9 (C-20), 135.0 (C-41), 132.1 (C-49), 130.6 (C-40), 130.3 (C-44), 130.3 (C-42), 130.2 (C-43), 127.6 (C-45), 114.4 (C-48), 113.8 (C-47), 109.4 (C-29), 96.5 (C-51), 78.0 (C-3), 69.6 (C-2), 55.3 (C-5), 54.6 (C-17), 53.7 (C-35, C-36, C-37, C-38, C-53), 52.5 (C-18), 51.2 (C-9), 45.7 (C-19), 42.2 (C-1), 42.0 (C-14), 40.7 (C-8), 37.4 (C-4), 37.0 (C-10), 36.8 (C-13), 35.8 (C-22), 34.2 (C-7), 32.5 (C-16), 31.2 (C-21), 29.6 (C-15), 28.9 (C-23), 25.4 (C-12), 21.2 (C-34), 20.8 (C-32), 20.8, 20.8 (C-11, C-54), 19.4 (C-30), 18.0 (C-6), 17.5 (C-25), 16.7 (C-26), 16.2 (C-24), 14.5 (C-27), 11.3 (C-55) ppm; MS (ESI, MeOH): *m*/*z* 1114.4 (100%, [M-Cl]^+^); analysis calcd for C_70_H_97_N_4_O_7_Cl (1142.02): C 73.62, H 8.56, N 4.91; found: C 73.44, H 8.71; N 4.67.

#### 4.2.19. 9-[2-[[4-(2β,3β)-Diacetoxy-lup-20(29)-en-28-oyl)-1-piperazinyl]carbonyl]phenyl]-3,6-bis(dibutylamino)-xanthylium Chloride (**16**) 

According to the GPC from **9** (260 mg, 0.42 mmol), followed by chromatography (SiO_2_, CHCl_3_/MeOH, 9:1), **16** (320 mg, 71%) was obtained as a violet solid; R_f_ = 0.53 (SiO_2_, CHCl_3_/MeOH, 9:1); m.p. = 265 °C; UV-Vis (MeOH): λ_max_ (log ε) = 560 nm (4.97); IR (ATR): ν = 2937*br*, 1737*w*, 1631*m*, 1586*vs*, 1528*w*, 1506*w*, 1466*m*, 1411*s*, 1335*s*, 1300*w*, 1272*w*, 1245*s*, 1179*vs*, 1131*m*, 1072*m*, 1031*w*, 1003*m*, 978*m*, 921*m*, 870*w*, 823*m*, 787*w*, 757*w*, 683*m*, 665*w*, 579*w*, 544*w*, 496*w*, 440*w* cm^−1^; ^1^H NMR (400 MHz, CDCl_3_): δ = 7.71–7.62 (*m*, 2H, 42-H, 43-H), 7.55–7.49 (*m*, 1H, 45-H), 7.38–7.32 (*m*, 1H, 44-H), 7.32–7.21 (*m*, 1H, 49-H), 7.05–6.92 (*m*, 1H, 47-H), 6.85–6.77 (*m*, 1H, 51-H), 5.30–5.26 (*m*, 1H, 2-H), 4.68–4.65 (*m*, 1H, 29-H_a_), 4.57 (*d*, *J* = 3.8 Hz, 1H, 3-H), 4.56–4.53 (*m*, 1H, 29-H_b_), 3.73–3.23 (*m*, 10H, 53-H, 35-H, 36-H, 37-H, 38-H), 2.88 (*dt*, *J* = 11.2, 6.1 Hz, 1H, 19-H), 2.78–2.68 (*m*, 1H, 13-H), 2.00 (*s*, 3H, 32-H), 1.99 (*s*, 3H, 34-H), 2.05–1.90 (*m*, 2H, 16-H_a_, 1-H_a_), 1.86–1.78 (*m*, 1H, 22-H_a_), 1.75–1.56 (*m*, 2H, 21-H_a_, 12-H_a_), 1.63 (*s*, 3H, 30-H), 1.56–1.43 (*m*, 4H, 6-H, 16-H_b_, 18-H), 1.42–1.17 (*m*, 12H, 7-H, 22-H_b_, 21-H_b_, 11-H, 54-H, 1-H_b_, 9-H, 15-H_a_), 1.17–1.02 (*m*, 1H, 15-H_b_, 12-H_b_), 1.06 (*s*, 3H, 26-H), 0.99 (*s*, 3H, 25-H), 0.90 (*s*, 4H, 27-H, 5-H), 0.88 (*s*, 3H, 24-H), 0.86 (*s*, 3H, 23-H) ppm; ^13^C NMR (100 MHz, CDCl_3_): δ = 174.3 (C-28), 170.8 (C-33), 170.4 (C-31), 167.8 (C-39), 157.9 (C-52), 155.8 (C-50), 155.7 (C-46), 151.0 (C-20), 135.1 (C-41), 132.4 (C-49), 130.8 (C-40), 130.5 (C-44), 130.4 (C-42), 130.3 (C-43), 127.7 (C-45), 114.5 (C-48), 114.0 (C-47), 109.6 (C-29), 96.6 (C-51), 78.1 (C-3), 69.7 (C-2), 55.4 (C-5), 54.8 (C-17), 52.6 (C-18), 51.3 (C-9), 46.4 (C-35, C-36, C-37, C-38), 46.4 (C-53), 45.8 (C-19), 42.3 (C-14), 42.2 (C-1), 40.9 (C-8), 37.5 (C-4), 37.1 (C-10), 37.0 (C-13), 36.0 (C-22), 34.3 (C-7), 32.6 (C-16), 31.3 (C-21), 29.8 (C-15), 29.1 (C-23), 25.6 (C-12), 21.4 (C-34), 21.3 (C-11), 21.0 (C-32), 19.6 (C-30), 18.1 (C-6), 17.6 (C-25), 16.9 (C-26), 16.3 (C-24), 14.6 (C-27), 12.8 (C-54) ppm; MS (ESI, MeOH): *m*/*z* 1050.7 (100%, [M-Cl]^+^); analysis calcd for C_74_H_105_N_4_O_7_Cl (1198.63): C 74.18, H 8.83, N 4.68; found: C 73.93, H 9.01; N 4.52.

#### 4.2.20. 9-[2-[[4-(2β,3β-Diacetoxy-lup-20(29)-en-28-oyl)-1-piperazinyl]carbonyl]phenyl)]-2,3,6,7,12,13,16,17-octahydro-1H,5H,11H,15H-xantheno(2,3,4-ij:5,6,7-i’j’)diquinolizin-18-ium Chloride (**17**)

According to the GPC from **9** (370 mg, 0.59 mmol), followed by chromatography (SiO_2_, CHCl_3_/MeOH, 9:1), **17** (296 mg, 44%) was obtained as a purple solid; R_f_ = 0.6 (SiO_2_, CHCl_3_/MeOH, 9:1); m.p. = 286 °C (decomp.); UV-Vis (MeOH): λ_max_ (log ε) = 582 nm (4.93); IR (ATR): ν = 2938*br*, 2864*w*, 1737*m*, 1630*m*, 1594*s*, 1542*w*, 1493*s*, 1459*m*, 1435*m*, 1361*m*, 1294*vs*, 1266*s*,1255*s*, 1181*s*, 1144*w*, 1099*s*, 1076*w*, 1034*m*, 1003*m*, 982*w*, 882*w*, 773*w*, 733*w*, 624*w*, 603*w*, 575*w*, 562*w*, 507*w* cm^−1^; ^1^H NMR (400 MHz, CDCl_3_): δ = 7.72–7.64 (*m*, 2H, 42-H, 43-H), 7.56–7.46 (*m*, 1H, 45-H), 7.35–7.28 (*m*, 1H, 44-H), 6.71–6.61 (*m*, 1H, 48-H), 5.32–5.25 (*m*, 1H, 2-H), 4.71–4.66 (*m*, 1H, 29-H_a_), 4.60–4.54 (*m*, 2H, 3-H, 29-H_b_), 3.65–3.24 (*m*, 12H, 55-H, 56-H, 35-H, 36-H, 37-H, 38-H), 3.07–2.96 (*m*, 2H, 53-H), 2.94–2.83 (*m*, 1H, 19-H), 2.81–2.60 (*m*, 3H, 13-H, 58-H), 2.20–1.86 (*m*, 6H, 54-H, 16-H_a_, 57-H, 1-H_a_), 2.01 (*s*, 3H, 32-H), 2.00 (*s*, 3H, 34-H), 1.85–1.58 (*m*, 3H, 22-H_a_, 21-H_a_, 12-H_a_), 1.65 (*s*, 3H, 30-H), 1.58–1.15 (*m*, 13H, 6-H, 16-H_b_, 18-H, 7-H, 22-H_b_, 21-H_b_, 11-H, 15-H_a_, 1-H_b_, 9-H), 1.15–1.09 (*m*, 1H, 15-H_b_), 1.08 (*s*, 3H, 25-H), 1.00 (*s*, 3H, 24-H), 0.92 (*s*, 3H, 27-H), 0.90 (*s*, 5H, 26-H, 12-H_b_, 5-H), 0.86 (*s*, 3H, 23-H) ppm; ^13^C NMR (100 MHz, CDCl_3_): δ = 174.2 (C-28), 170.8 (C-33), 170.4 (C-31), 168.0 (C-39), 152.1 (C-52), 151.4 (C-46), 150.9 (C-20), 134.9 (C-40), 132.0 (C-41), 130.9 (C-44), 130.4 (C-42), 129.9 (C-43), 127.6 (C-45), 126.6 (C-48), 123.7 (C-49), 113.3 (C-47), 109.7 (C-29), 105.6 (C-51), 78.1 (C-3), 69.7 (C-2), 55.4 (C-5), 54.8 (C-17), 52.6 (C-18), 51.3 (C-9), 51.1 (C-56), 50.7 (C-55), 47.7 (C-36, C-38), 45.8 (C-19), 42.3 (C-14), 42.2 (C-1), 42.0 (C-35, C-37), 40.9 (C-8), 37.5 (C-4), 37.1 (C-10), 37.0 (C-13), 36.0 (C-22), 34.4 (C-7), 32.7 (C-16), 31.4 (C-21), 29.8 (C-15), 29.1 (C-23), 25.6 (C-12), 21.4 (C-34), 21.4 (C-11), 21.0 (C-32), 20.8 (C-57), 20.0 (C-53), 19.8 (C-54), 19.6 (C-30), 18.1 (C-6), 17.6 (C-24), 16.9 (C-25), 16.3 (C-26), 14.7 (C-27) ppm; MS (ESI, MeOH/CHCl_3_): *m*/*z* 1098.4 (100%, [M-Cl]^+^); analysis calcd for C_70_H_89_N_4_O_7_Cl (1133.95): C 74.15, H 7.91, N 4.94; found: C 73.96, H 8.13; N 4.77.

#### 4.2.21. 9-[2-[[4-(2β,3β-Diacetoxy-lup-20(29)-en-28-oyl)-1 -(exahydro-1H-1,4-diazepin-1-yl)]carbonyl]phenyl]-3,6-bis(dimethylamino)-xanthylium Chloride (**18**)

According to the GPD from **10** (220 mg, 0.34 mmol), followed by chromatography (SiO_2_, CHCl_3_/MeOH, 9:1), **18** (252 mg, 70%) was obtained as a violet solid; R_f_ = 0.56 (SiO_2_ CHCl_3_/MeOH, 9:1); m.p. = 274 °C (decomp.); UV-Vis (MeOH): λ_max_ (log ε) = 557 nm (4.93); IR (ATR): ν = 2935*w*, 1738*m*, 1591*s*, 1493*m*, 1407*m*, 1342*s*, 1251*m*, 1184*s*, 1133*m*, 1031*w*, 925*m*, 820*w*, 699*m*, 516*m* cm^−1^; ^1^H NMR (500 MHz, CDCl_3_): δ = 7.67–7.56 (*m*, 2H, 44-H, 45-H), 7.44–7.39 (*m*, 1H, 46-H), 7.36–7.29 (*m*, 1H, 43-H), 7.29–7.16 (*m*, 1H, 50-H), 7.13–6.89 (*m*, 1H, 49-H), 6.87–6.73 (*m*, 1H, 52-H), 5.29–5.23 (*m*, 1H, 2-H), 4.73–4.65 (*m*, 1H, 29-H_a_), 4.61–4.50 (*m*, 2H, 3-H, 29-H_b_), 3.37–3.27 (*m*, 6H, 54-H), 3.00–2.90 (*m*, 1H, 19-H), 2.87–2.76 (*m*, 1H, 13-H), 2.12–2.03 (*m*, 1H, 16-H_a_), 1.99 (*s*, 3H, 32-H), 1.97 (*s*, 3H, 34-H), 1.97–1.83 (*m*, 2H, 1-H_a_, 22-H_a_), 1.81–1.65 (*m*, 2H, 21-H_a_, 12-H_a_), 1.62 (*s*, 3H, 30-H), 1.55–1.40 (*m*, 4H, 6-H, 18-H, 16-H_b_), 1.39–1.14 (*m*, 10H, 21-H_b_, 11-H, 7-H, 22-H_b_, 15-H, 1-H_b_, 9-H), 1.06 (*s*, 3H, 25-H), 0.98 (*s*, 3H, 24-H), 0.90 (*s*, 5H, 26-H, 12-H_b_, 5-H), 0.87 (*s*, 3H, 27-H), 0.83 (*s*, 3H, 23-H) ppm; ^13^C NMR (125 MHz, CDCl_3_): δ = 170.7 (C-33), 170.2 (C-31), 168.6 (C-40), 157.5 (C-53), 157.3 (C-51), 151.3 (C-47), 151.0 (C-20), 136.2 (C-42), 132.2 (C-50), 130.1 (C-43), 130.0 (C-44), 129.6 (C-45), 126.7 (C-46), 114.2 (C-49), 113.7 (C-48), 109.3 (C-29), 96.9 (C-52), 78.0 (C-3), 69.6 (C-2), 55.3 (C-5), 55.0 (C-17), 52.9 (C-18), 51.2 (C-9), 45.8 (C-19), 42.2 (C-14), 42.1 (C-1), 41.2 (C-54), 40.8 (C-8), 37.4 (C-4), 37.0 (C-10), 36.7 (C-13), 36.1 (C-22), 34.3 (C-7), 32.4 (C-16), 31.4 (C-21), 29.6 (C-15), 28.9 (C-23), 25.5 (C-12), 21.3 (C-11), 21.2 (C-32), 20.8 (C-34), 19.5 (C-30), 18.0 (C-6), 17.5 (C-24), 16.7 (C-25), 16.3 (C 26), 14.6 (C-27) ppm; MS (ESI, MeOH/CHCl_3_): *m*/*z* 1007.2 (100%, [M-Cl]^+^); analysis calcd for C_63_H_83_N_4_O_7_Cl (1043.83): C 72.49, H 8.02, N 3.40; found: C 72.19, H 8.26; N 3.17.

#### 4.2.22. 9-[2-[[4-(2β,3β-Diacetoxy-lup-20(29)-en-28-oyl)-1-(hexahydro-1H-1,4-diazepin-1-yl)]carbonyl]phenyl]-3,6-bis(diethylamino)-xanthylium Chloride (**19**)

According to the GPD from **10** (400 mg, 0.63 mmol), followed by chromatography (SiO_2_, CHCl_3_/MeOH, 9:1), **19** (485 mg, 70%) was obtained as a violet solid; R_f_ = 0.4 (SiO_2_, CHCl_3_/MeOH 9:1); m.p. = 264 °C (decomp.); UV-Vis (MeOH): λ_max_ (log ε) = 562 nm (4.98); IR (ATR): ν = 2936*br*, 2869*w*, 1738*m*, 1625*m*, 1586*vs*, 1528*w*, 1481*w*, 1467*m*, 1411*s*, 1377*w* 1335*s*, 1272*w*, 1245*s*, 1179*vs*, 1132*m*, 1095*w*, 1072*m*, 1031*w*, 1010*w*, 978*w*, 921*m*, 870*w*, 822*m*, 788*w*, 754*m*, 683*m*, 665*w*, 629*w*, 620*w*, 602*w*, 579*w*, 497*w* cm^−1^; ^1^H-NMR (400 MHz, CDCl_3_): δ = 7.67–7.56 (*m*, 2H, 45-H, 44-H), 7.43 (*d*, *J* = 7.3 Hz, 1H, 46-H), 7.33–7.17 (*m*, 2H, 43-H, 50-H), 6.84–6.70 (*m*, 1H, 52-H), 5.30–5.25 (*m*, 1H, 2-H), 4.74–4.66 (*m*, 1H, 29-H_a_), 4.60–4.53 (*m*, 2H, 29-H_b_, 3-H), 3.93–3.15 (*m*, 12H, 35-H, 36-H, 37-H, 38-H, 39-H, 54-H), 2.96 (*d*, *J* = 19.0 Hz, 1H, 19-H), 2.90–2.78 (*m*, 1H, 13-H), 2.00 (*s*, 3H, 32-H), 1.98 (*s*, 3H, 34-H), 2.06–1.84 (*m*, 3H, 16-H_a_, 1-H_a_, 22-H_a_), 1.83–1.56 (*m*, 6H, 21-H_a_, 12-H, 30-H), 1.56–1.16 (*m*, 15H, 6-H, 18-H, 16-H_b_, 21-H_b_, 7-H, 22-H_b_, 11-H, 55-H, 15-H_a_, 1-H_b_, 9-H), 1.15–1.03 (*m*, 1H, 15-H_b_), 1.07 (*s*, 3H, 25-H), 0.99 (*s*, 3H, 24-H), 0.91 (*s*, 4H, 26-H, 5-H), 0.89 (*s*, 3H, 27-H), 0.85 (*s*, 3H, 23-H) ppm; ^13^C-NMR (100 MHz, CDCl_3_): δ = 175.7 (C-28), 170.8 (C-33), 170.4 (C-31), 168.4 (C-40), 157.8 (C-53), 156.0 (C-47), 155.8 (C-51), 155.7 (C-20), 136.1 (C-42), 131.9 (C-50), 130.7 (C-41), 130.3 (C-43), 130.0 (C-44), 129.7 (C-45), 126.8 (C-46), 113.9 (C-49), 113.7 (C-48), 109.3 (C-29), 96.6 (C-52), 78.1 (C-3), 69.7 (C-2), 55.5 (C-5), 55.1 (C-17), 53.1 (C-18), 51.4 (C-9), 46.4 (C-39), 46.3 (C-35, C-36, C-37, C-38, C-54), 46.0 (C-19), 42.3 (C-1), 42.2 (C-14), 40.9 (C-8), 37.5 (C-4), 37.1 (C-10), 36.9 (C-13), 36.1 (C-22), 34.4 (C-7), 32.0 (C-16), 31.6 (C-21), 29.9 (C-15), 29.0 (C-23), 25.6 (C-12), 21.4 (C-11), 21.3 (C-32), 21.0 (C-34), 19.6 (C-30), 18.1 (C-6), 17.6 (C-24), 16.9 (C-25), 16.4 (C-26), 14.7 (C-27), 12.8 (C-55) ppm; MS (ESI, MeOH): *m*/*z* 1064.4 (100%, [M-Cl]^+^); analysis calcd for C_67_H_91_N_4_O_7_Cl (1063.69): C 75.60, H 8.62, N 5.26; found: C 75.36, H 8.91, N 5.07.

#### 4.2.23. 9-[2-[[4-(2β,3β-Diacetoxy-lup-20(29)-en-28-oyl)-1-(hexahydro-1H-1,4-diazepin-1-yl)]carbonyl]phenyl]-3,6-bis(dipropylamino)-xanthylium Chloride (**20**)

According to the GPD from **10** (250 mg, 0.39 mmol), followed by chromatography (SiO_2_, CHCl_3_/MeOH, 9:1), **20** (264 mg, 60%) was obtained as a purple solid; R_f_ = 0.61 (SiO_2_, CHCl_3_/MeOH, 9:1); m.p. = 212 °C; UV-Vis (MeOH): λ_max_ (log ε) = 565 nm (4.98); IR (ATR): ν = 2935*br*, 2872*w*, 1739*m*, 1625*m*, 1587*vs*, 1527*w*, 1507*w*, 1468*m*, 1411*m*, 1376*w*, 1337*s*, 1300*m*,1251*m*, 1230*s*, 1177*vs*, 1132*m*, 1099*m*, 1074*w*, 1031*w*, 981*w*, 939*m*, 917*w*, 877*w*, 822*m*, 779*w*, 757*w*, 706*w*, 665*w*, 599*w*, 574*w*, 562*w*, 507*w*, 457*w* cm^−1^; ^1^H NMR (500 MHz, CDCl_3_): δ = 7.66–7.55 (*m*, 2H, 44-H, 43-H), 7.42 (*d*, *J* = 6.7 Hz, 1H, 46-H), 7.32–7.15 (*m*, 2H, 45-H, 50-H), 6.95–6.77 (*m*, 1H, 49-H), 6.77–6.64 (*m*, 1H, 52-H), 5.30–5.25 (*m*, 1H, 2-H), 4.70 (*d*, *J* = 10.7 Hz, 1H, 29-H_a_), 4.61–4.49 (*m*, 2H, 3-H, 29-H_b_), 3.63–3.29 (*m*, 12H, 54-H, 35-H, 36-H, 37-H, 38-H, 39-H), 3.02–2.90 (*m*, 1H, 19-H), 2.88–2.78 (*m*, 1H, 13-H), 2.13–2.06 (*m*, 1H, 16-H_a_), 1.99 (*s*, 3H, 32-H), 1.98 (*s*, 3H, 34-H), 1.97–1.84 (*m*, 2H, 1-H_a_, 22-H_a_), 1.80–1.64 (*m*, 5H, 21-H_a_, 55-H, 12-H_a_, 11-H_a_), 1.63 (*s*, 3H, 30-H), 1.56–1.41 (*m*, 4H, 6-H, 18-H, 16-H_b_), 1.40–1.17 (*m*, 9H, 21-H_b_, 7-H, 11-H_b_, 22-H_b_, 15-H, 1-H_b_, 9-H), 1.06 (*s*, 3H, 26-H), 0.98 (*s*, 6H, 56-H, 25-H), 0.90 (*s*, 4H, 24-H, 12-H_b_), 0.88 (*s*, 4H, 27-H, 5-H), 0.84 (*s*, 3H, 23-H) ppm; ^13^C NMR (125 MHz, CDCl_3_): δ = 170.8 (C-33), 170.3 (C-31), 157.8 (C-53), 156.2 (C-47), 156.1 (C-51), 151.4 (C-20), 135.2 (C-42), 131.3 (C-50), 130.3 (C-45), 130.0 (C-43), 129.7 (C-44), 126.8 (C-46), 114.5 (C-49), 113.5 (C-48), 109.0 (C-29), 96.4 (C-52), 78.1 (C-3), 69.7 (C-2), 55.4 (C-5), 55.1 (C-17), 53.9 (C-39, C-54), 53.8 (C-35, C-36, C-37, C-38), 52.7 (C-18), 51.3 (C-9), 45.9 (C-19), 42.3 (C-14), 42.2 (C-1), 40.9 (C-8), 37.5 (C-4), 37.1 (C-10), 36.8 (C-13), 36.2 (C-22), 34.3 (C-7), 32.7 (C-16), 31.6 (C-21), 29.8 (C-15), 29.0 (C-23), 25.6 (C-12), 21.4 (C-11), 21.3 (C-34), 20.9 (C-32), 20.9 (C-55), 19.6 (C-30), 18.1 (C-6), 17.6 (C-25), 16.8 (C-26), 16.3 (C-24), 14.6 (C-27), 11.4 (C-56) ppm; MS (ESI, MeOH/CHCl_3_): *m*/*z* 1120.1 (100%, [M-Cl]^+^); analysis calcd for C_71_H_99_N_4_O_7_Cl (1156.04): C 73.77, H 8.63, N 4.85; found: C 73.54, H 8.90; N 4.61.

#### 4.2.24. 9-[2-[[4-(2β,3β-Diacetoxy-lup-20(29)-en-28-oyl)-1-(hexahydro-1H-1,4-diazepin-1-yl)]carbonyl]phenyl]-3,6-bis(dibutylamino)-xanthylium Chloride (**21**)

According to the GPC from **10** (250 mg, 0.39 mmol), followed by chromatography (SiO_2_, CHCl_3_/MeOH, 9:1), **21** (244 mg, 0.2 mmol, 52%) was obtained as a purple solid; R_f_ = 0.61 (SiO_2_, CHCl_3_/MeOH, 9:1); m.p. = 216 °C; UV-Vis (MeOH): λ_max_ (log ε) = 568 nm (5.04); IR (ATR): ν = 2932*br*, 2869*w* 1739*m*, 1625*m*, 1586*vs*, 1528*w*, 1507*w*, 1461*m*, 1411*s*, 1339*s*, 1291*w*, 1250*m*, 1218*s*, 1175*vs*, 1132*m*, 1109*w*, 1054*w*, 1031*w*, 982*w*, 921*m*, 881*w*, 822*m*, 755*w*, 704*m*, 664*w*, 601*w*, 569*w*, 509*w*, 488*w*, 461*w* cm^−1^; ^1^H NMR (400 MHz, CDCl_3_): δ = 7.67–7.55 (*m*, 2H, 43-H, 44-H), 7.46–7.42 (*m*, 1H, 46-H), 7.33–7.17 (*m*, 2H, 50-H, 45-H), 6.96–6.78 (*m*, 1H, 49-H), 6.78–6.66 (*m*, 1H, 52-H), 5.29 (*d*, *J* = 4.3 Hz, 1H, 2-H), 4.75–4.68 (*m*, 1H, 29-H_a_), 4.61–4.54 (*m*, 2H, 3-H, 29-H_b_), 3.90–3.23 (*m*, 12H, 35-H, 36-H, 37-H, 38-H, 39-H, 54-H), 3.05–2.93 (*m*, 1H, 19-H), 2.91–2.78 (*m*, 1H, 13-H), 2.17–2.05 (*m*, 1H, 16-H_a_), 2.01 (*s*, 3H, 32-H), 2.00 (*s*, 3H, 34-H), 1.99–1.87 (*m*, 2H, 1-H_a_, 22-H_a_), 1.85–1.65 (*m*, 4H, 21-H_a_, 12-H_a_, 55-H), 1.65 (*s*, 3H, 30-H), 1.57–1.15 (*m*, 15H, 6-H, 18-H, 56-H, 21-H_b_, 11-H, 7-H, 22-H_b_, 15-H_a_, 1-H_b_, 9-H), 1.14–1.11 (*m*, 1H, 15-H_b_), 1.08 (*s*, 3H, 26-H), 1.00 (*s*, 3H, 25-H), 0.98 (*s*, 3H, 57-H), 0.92 (*s*, 4H, 24-H, 12-H_b_), 0.90 (*s*, 4H, 27-H, 5-H), 0.86 (*s*, 3H, 23-H) ppm; ^13^C NMR (100 MHz, CDCl_3_): δ = 175.6 (C-28), 170.8 (C-33), 170.4 (C-31), 168.8 (C-40), 157.7 (C-53), 156.2 (C-47), 156.1 (C-51), 151.2 (C-20), 136.2 (C-42), 131.9 (C-50), 130.3 (C-45), 130.1 (C-44), 129.6 (C-43), 126.9 (C-46), 114.5 (C-49), 113.8 (C-48), 109.4 (C-29), 96.6 (C-52), 78.1 (C-3), 69.8 (C-2), 55.5 (C-5), 55.2 (C-17), 53.1 (C-18), 52.1 (C-35, C-36, C-37, C-38, C-39, C-54), 51.4 (C-9), 46.0 (C-19), 42.3 (C-14), 42.2 (C-1), 40.9 (C-8), 37.5 (C-4), 37.1 (C-10), 36.9 (C-13), 36.0 (C-22), 34.4 (C-7), 32.1 (C-16), 31.6 (C-21), 29.7 (C-15), 29.6 (C-55), 29.1 (C-23), 25.7 (C-12), 21.4 (C-11), 21.4 (C-34), 21.0 (C-32), 20.3 (C-56), 19.5 (C-30), 18.1 (C-6), 17.6 (C-25), 16.9 (C-26), 16.4 (C-24), 14.7 (C-27), 14.0 (C-57) ppm; MS (ESI, MeOH/CHCl_3_): *m*/*z* 1176.3 (100%, [M-Cl]^+^); analysis calcd for C_75_H_107_N_4_O_7_Cl (1212.15): C 74.32, H 8.90, N 4.62; found: C 74.08, H 9.16; N 4.46.

#### 4.2.25. 9-[2-[[4-(2β,3β-Diacetoxy-lup-20(29)-en-28-oyl)-1-(hexahydro-1H-1,4-diazepin-1-yl)]carbonyl]phenyl)]-2,3,6,7,12,13,16,17-octahydro-1H,5H,11H,15H-xantheno(2,3,4-ij:5,6,7-i’j’)diquinolizin-18-ium Chloride (**22**)

According to the GPC from **10** (277 mg, 0.43 mmol), followed by chromatography (SiO_2_, CHCl_3_/MeOH, 9:1), **22** (332 mg, 66%) was obtained as a purple solid; R_f_ = 0.34 (SiO_2_, CHCl_3_/MeOH, 9:1); m.p. = 290 °C (decomp.); UV-Vis (MeOH): λ_max_ (log ε) = 583 nm (4.90); IR (ATR): ν = 2939*br*, 1736*m*, 1595*s*, 1493*s*, 1361*m*, 1294*vs*, 1181*s*, 1099*s*, 1034*m*, 420*s* cm^−1^; ^1^H NMR (500 MHz, CDCl_3_): δ = 7.70–7.54 (*m*, 2H; 43-H, 44-H), 7.48–7.39 (*m*, 1H, 46-H), 7.33–7.18 (*m*, 1H, 45-H), 6.81–6.61 (*m*, 1H, 49-H), 5.34–5.24 (*m*, 1H, 2-H), 4.79–4.66 (*m*, 1H, 29-H_a_), 4.64–4.53 (*m*, 2H, 3-H, 29-H_b_), 3.71–3.38 (*m*, 4H, 56-H, 57-H), 3.07–2.94 (*m*, 3H, 54-H, 19-H), 2.94–2.79 (*m*, 1H, 13-H), 2.78–2.63 (*m*, 2H, 59-H), 2.17–2.04 (*m*, 3H, 55-H, 16-H_a_), 2.01 (*s*, 3H, 32-H), 2.00 (*s*, 3H, 34-H), 1.99–1.85 (*m*, 4H, 58-H, 1-H_a_, 22-H_a_), 1.85–1.61 (*m*, 2H, 12-H_a_, 21-H_a_), 1.65 (*s*, 3H, 30-H), 1.59–1.43 (*m*, 4H, 6-H, 16-H_b_, 18-H), 1.43–1.18 (*m*, 9H, 21-H_b_, 7-H, 11-H, 22-H_b_, 15-H_a_, 1-Hb, 9-H), 1.16–1.10 (*m*, 1H, 15-H_b_), 1.08 (*s*, 3H, 25-H), 1.00 (*s*, 3H, 24-H), 0.97–0.88 (*m*, 8H, 26-H, 27-H, 12-H_b_, 5-H), 0.86 (*s*, 3H, 23-H) ppm; ^13^C NMR (125 MHz, CDCl_3_): δ = 174.8 (C-28), 170.8 (C-33), 170.4 (C-31), 152.1 (C-53), 151.4 (C-47), 151.1 (C-20), 132.0 (C-42), 130.6 (C-45), 130.0 (C-43), 129.7 (C-44), 127.8 (C-46), 126.6 (C-49), 113.5 (C-48), 109.5 (C-29), 105.4 (C-52), 78.1 (C-3), 69.7 (C-2), 55.5 (C-5), 55.2 (C-17), 52.8 (C-18), 51.4 (C-9), 51.1 (C-57), 50.7 (C-56), 45.7 (C-19), 42.4 (C-14), 42.2 (C-1), 40.9 (C-8), 37.5 (C-4), 37.1 (C-10), 36.9 (C-13), 36.2 (C-22), 34.4 (C-7), 32.2 (C-16), 31.5 (C-21), 29.9 (C-15), 29.1 (C-23), 27.8 (C-59), 25.6 (C-12), 21.4 (C-11), 21.4 (C-34), 21.0 (C-32), 20.8 (C-58), 20.1 (C-54), 19.9 (C-55), 19.6 (C-30), 18.1 (C-6), 17.6 (C-24), 16.9 (C-25), 16.3 (C-26), 14.7 (C-27) ppm; MS (ESI, MeOH): *m*/*z* 1111.0 (100%, [M-Cl]^+^); analysis calcd for C_71_H_91_N_4_O_7_Cl (1147.98): C 74.29, H 7.99, N 4.88; found: C 74.03, H 8.18; N 4.67.

#### 4.2.26. 9-[2-[[4-(2β,3β-Diacetoxy-20-oxo-30-norlupan-28-oyl)-1-piperazinyl]carbonyl]phenyl]-3,6-bis(dimethylamino)-xanthylium Chloride (**23**)

According to the GPC from **11** (175 mg, 0.27 mmol), followed by chromatography (SiO_2_, CHCl_3_/MeOH, 9:1), **23** (180 mg, 63%) was obtained as a purple solid; R_f_ = 0.55 (SiO_2_, CHCl_3_/MeOH 9:1); m.p.: = 310–314 °C; UV-Vis (MeOH): λ_max_ (log ε) = 556 nm (4.94); IR (ATR): ν = 2937*br*, 1736*m*, 1591*vs*, 1534*w*, 1493*m*, 1407*s*, 1364*s*, 1343vs, 1259*m*, 1185*vs*, 1134*m*, 1031*w*, 1003*m*, 926*m*, 821*w*, 699*m*, 580*w*, 517*w* cm^−1^; ^1^H-NMR (500 MHz, CDCl_3_): δ = 7.71–7.62 (*m*, 2H, 42-H, 43-H), 7.54–7.49 (*m*, 1H, 44-H), 7.38–7.29 (*m*, 1H, 41-H), 7.27–7.20 (*m*, 1H, 48-H), 7.09–6.99 (*m*, 1H, 47-H), 6.90–6.80 (*m*, 1H, 50-H), 5.30–5.25 (*m*, 1H, 2-H), 4.55 (*d*, *J* = 3.8 Hz, 2H, 3-H), 3.51–3.21 (*m*, 20H, 53-H, 34-H, 35-H, 36-H, 37-H), 3.16–3.07 (*m*, 1H, 19-H), 2.55 (*td*, *J* = 12.5, 3.5 Hz, 1H, 13-H), 2.10 (*s*, 3H, 29-H), 2.03–1.98 (*m*, 1H, 18-H), 1.99 (*s*, 3H, 33-H), 1.98 (*s*, 3H, 31-H), 2.01–1.91 (*m*, 2H, 16-H_a_, 1-H_a_), 1.90–1.77 (*m*, 2H, 22-H_a_, 21-H_a_), 1.63–1.15 (*m*, 12H, 16-H_b_, 6-H, 22-H_b_, 21-H_b_, 7-H, 11-H, 9-H, 15-H_a_, 1-H_b_), 1.14–1.07 (*m*, 1H, 15-H_b_), 1.05 (*s*, 3H, 26-H), 0.98 (*s*, 3H, 25-H), 1.02–0.93 (*m*, 2H, 12-H), 0.91 (*s*, 3H, 27-H), 0.88–0.86 (*m*, 1H, 5-H), 0.85 (*s*, 3H, 24-H), 0.84 (*s*, 3H, 23-H) ppm; ^13^C-NMR (125 MHz, CDCl_3_): δ = 212.7 (C-20), 174.2 (C-28), 170.7 (C-32), 170.3 (C-30), 167.7 (C-38), 157.6 (C-51), 157.5 (C-49), 156.1 (C-45), 135.1 (C-40), 132.0 (C-48), 130.7 (C-39), 130.4 (C-43), 130.4 (C-42), 130.3 (C-41), 127.7 (C-44), 114.7 (C-47), 114.0 (C-46), 97.0 (C-50), 78.0 (C-3), 69.7 (C-2), 55.4 (C-5), 54.6 (C-17), 52.5 (C-18), 51.2 (C-9), 50.1 (C-19), 42.2 (C-1), 42.0 (C-14), 41.4 (C-52, C-34, C-35, C-36, C-37), 40.7 (C-8), 37.5 (C-4), 37.1 (C-10), 35.9 (C-13), 35.7 (C-22), 34.2 (C-7), 32.1 (C-16), 30.2 (C-29), 29.8 (C-15), 29.0 (C-23), 28.8 (C-21), 27.4 (C-12), 21.3 (C-33), 21.3 (C-11), 18.0 (C-6), 17.6 (C-25), 16.8 (C-26), 16.2 (C-24), 14.6 (C-27) ppm; MS (ESI, MeOH): *m*/*z* 996.5 (100%, [M-Cl]^+^); analysis calcd for C_61_H_79_N_4_O_8_Cl (995.59): C 73.54, H 7.99, N 5.62; found: C 73.81, H 8.39, N 4.32.

#### 4.2.27. 9-[2-[[4-(2β,3β-Diacetoxy-20-oxo-30-norlupan-28-oyl)-1-piperazinyl]carbonyl]phenyl]-3,6-bis(diethylamino)-xanthylium Chloride (**24**) 

According to the GPC from **11** (500 mg, 0.8 mmol), followed by chromatography (SiO_2_, CHCl_3_/MeOH, 9:1), **24** (504 mg, 59%) was obtained as a purple solid; R_f_ = 0.35 (SiO_2_, CHCl_3_/MeOH, 9:1); m.p. = 265–269 °C; UV-Vis (MeOH): λ_max_ (log ε) = 561 nm (5.02); IR (ATR): ν = 2934*w*, 1736*m*, 1630*m*, 1586*vs*, 1528*w*, 1466*m*, 1411*s*, 1335*s*, 1245*s*, 1177*vs*, 1131*s*, 1072*s*, 1003*s*, 921*m*, 822*m*, 683*s*, 497*w* cm^−1^; ^1^H NMR (500 MHz, CDCl_3_): δ = 7.70–7.62 (*m*, 2H, 42-H, 43-H), 7.54–7.48 (*m*, 1H, 44-H), 7.35–7.29 (*m*, 1H, 41-H), 7.26–7.29 (*m*, 2H, 48-H), 7.09–6.92 (*m*, 2H, 47-H), 6.82–6.76 (*m*, 2H, 50-H), 3.73–3.52 (*m*, 1H, 2-H), 4.54 (*d*, *J* = 3.7 Hz, 1H, 3-H), 3.73–3.52 (*m*, 16H, 37-H, 36-H, 35-H, 34-H, 52-H), 3.10 (*td*, *J* = 11.2, 3.6 Hz, 1H, 19-H), 2.55 (*td*, *J* = 12.5, 3.5 Hz, 1H, 13-H), 2.09 (*s*, 3H, 29-H), 2.04–2.00 (*m*, 1H, 18-H), 1.98 (*s*, 3H, 33-H), 1.97 (*s*, 3H, 31-H), 2.05–1.88 (*m*, 2H, 16-H_a_, 1-H_a_), 1.89–1.70 (*m*, 2H, 22-H_a_, 21-H_a_), 1.64–1.37 (*m*, 5H, 16-H_b_, 6-H, 22-H_b_, 21-H_b_), 1.29 (*dd*, *J* = 14.5, 6.8 Hz, 12H, 53-H), 1.37–1.14 (*m*, 7H, 7-H, 11-H, 19-H, 15-H_a_, 1-H_b_), 1.04 (*s*, 3H, 25-H), 1.13–0.87 (*m*, 3H, 15-H_b_, 12-H), 0.98 (*s*, 3H, 24-H), 0.90 (*s*, 3H, 27-H), 0.84 (*s*, 3H, 26-H), 0.83 (*s*, 3H, 23-H), 0.80–0.77 (*m*, 1H, 5-H) ppm; ^13^C NMR (101 MHz, CDCl_3_): δ = 212.6 (C-20), 174.0 (C-28), 170.7 (C-32), 170.3 (C-30), 167.7 (C-38), 157.8 (C-51), 155.8 (C-45), 155.7 (C-49), 135.0 (C-40), 132.2 (C-48), 130.9 (C-39), 130.4 (C-41), 130.3 (C-42, C-43), 127.7 (C-44), 114.5 (C-47) 113.8 (C-46), 96.5 (C-50), 78.0 (C-3), 69.6 (C-2), 55.3 (C-5), 54.6 (C-17), 52.4 (C-18), 51.1 (C-9), 50.1 (C-19), 46.3 (C-37, C-36, C-35, C-34, C-52), 42.2 (C-1), 41.9 (C-14), 40.7 (C-8), 37.4 (C-4), 37.1 (C-10), 35.9 (C-13), 35.7 (C-22), 34.1 (C-7), 32.1 (C-16), 30.2 (C-29), 29.7 (C-15), 29.0 (C-23), 28.7 (C-21), 27.3 (C-12), 21.3 (C-33), 21.3 (C-11), 20.9 (C-31), 18.0 (C-6), 17.5 (C-24), 16.8 (C-25), 16.2 (C-26), 14.6 (C-27), 12.8 (C-53) ppm; MS (ESI, MeOH/CHCl_3_): *m*/*z* 1052.4 (100%, [M-Cl]^+^); analysis calcd for C_65_H_87_N_4_O_8_Cl (1087.88): C 71.76, H 8.06, N 5.15; found: C 71.54, H 8.29; N 4.97.

#### 4.2.28. 9-[2-[[4-(2β,3β-Diacetoxy-20-oxo-30-norlupan-28-oyl)-1-piperazinyl]carbonyl]phenyl]-3,6-bis(dipropylamino)-xanthylium Chloride (**25**) 

According to the GPC from **11** (175 mg, 0.28 mmol), followed by chromatography (SiO_2_, CHCl_3_/MeOH, 9:1), **25** (145 mg, 46%) was obtained as a purple solid; R_f_ = 0.52 (SiO_2_, CHCl_3_/MeOH, 9:1); m.p. = 204–208 °C; UV-Vis (MeOH): λ_max_ (log ε) = 564 nm (5.04); IR (ATR): ν = 2935*br*, 1739*m*, 1633*m*, 1587*vs*, 1469*m*, 1412*m*, 1337*s*, 1301*w*, 1231*s*, 1177*vs*, 1132*m*, 1100*m*, 1031*m*, 1003*m*, 940*w*, 823*w*, 756*w*, 706*w*, 666*w*, 563*w*, 507*w* cm^−1^; ^1^H NMR (500 MHz, CDCl_3_): δ = 7.71–7.64 (*m*, 2H, 42-H, 43-H), 7.57–7.51 (*m*, 1H, 44-H), 7.38–7.33 (*m*, 1H, 41-H), 7.32–7.21 (*m*, 1H, 48-H), 7.07–6.95 (*m*, 1H, 47-H), 6.82–6.69 (*m*, 1H, 50-H), 5.29 (*dd*, *J* = 3.7 Hz, 1H, 2-H), 4.58 (*d*, *J* = 3.7 Hz, 1H, 3-H), 3.68–3.27 (m, 16H, 52-H, 34-H, 35-H, 36-H, 37-H), 3.20–3.09 (*m*, 1H, 19-H), 2.58 (*td*, *J* = 12.3, 3.0 Hz, 1H, 13-H), 2.12 (*s*, 3H, 29-H), 2.06–1.92 (*m*, 3H, 18-H, 16-H_a_, 1-H_a_), 2.01 (*s*, 3H, 33-H), 2.00 (*s*, 3H, 31-H), 1.92–1.81 (*m*, 2H, 22-H_a_, 21-H_a_), 1.80–1.66 (*m*, 8H, 53-H), 1.65–1.18 (*m*, 12H, 16-H_b_, 6-H, 22-H_b_, 21-H_b_, 7-H, 11-H, 15-H_a_, 1-H_b_, 9-H), 1.16–1.09 (*m*, 1H, 15-H_b_), 1.07 (*s*, 3H, 26-H), 1.04–0.98 (*m*, 17H, 25-H, 54-H, 12-H), 0.93 (*s*, 3H, 27-H), 0.92–0.91 (*m*, 1H, 5-H), 0.88 (*s*, 3H, 24-H), 0.86 (*s*, 3H, 23-H) ppm; ^13^C NMR (126 MHz, CDCl_3_): δ = 212.7 (C-20), 174.2 (C-28), 170.8 (C-32), 170.3 (C-30), 167.8 (C-38), 157.8 (C-51), 156.2 (C-49), 155.7 (C-45), 135.1 (C-40), 132.2 (C-48), 130.9 (C-39), 130.6 (C-43), 130.4 (C-42), 130.4 (C-41), 127.8 (C-44), 114.5 (C-47), 114.0 (C-46), 96.6 (C-50), 78.0 (C-3), 69.7 (C-2), 55.4 (C-5), 54.7 (C-17), 53.9 (C-52), 52.6 (C-18), 51.2 (C-9), 50.2 (C-19), 45.1 (C-34, C-35, C-36, C-37), 42.3 (C-14), 42.0 (C-1), 40.8 (C-8), 37.5 (C-4), 37.1 (C-10), 36.0 (C-13), 35.8 (C-22), 34.2 (C-7), 32.2 (C-16), 30.2 (C-29), 29.8 (C-15), 29.1 (C-23), 28.8 (C-21), 27.4 (C-12), 21.4 (C-11), 21.4 (C-33), 21.0 (C-31), 20.9 (C-53), 18.1 (C-6), 17.6 (C-25), 16.9 (C-26), 16.3 (C-24), 14.7 (C-27), 11.5 (C-54) ppm; MS (ESI, MeOH): *m*/*z* 1108.6 (100%, [M-Cl]^+^); analysis calcd for C_70_H_97_N_4_O_7_Cl (1142.02): C 73.62, H 8.56, N 4.91; found: C 73.41, H 8.78; N 4.75.

#### 4.2.29. 9-[2-[[4-(2β,3β-Diacetoxy-20-oxo-30-norlupan-28-oyl)-1-piperazinyl]carbonyl]phenyl]-2,3,6,7,12,13,16,17-octahydro-1H,5H,11H,15H-xantheno(2,3,4-ij:5,6,7-i’j’)diquinolizin-18-ium Chloride (**26**)

According to the GPD from **11** (80 mg, 0.13 mmol), followed by chromatography (SiO_2_, CHCl_3_/MeOH, 9:1), **26** (102 mg, 69%) was obtained as a purple solid; R_f_ = 0.3 (SiO_2_, CHCl_3_/MeOH, 9:1); m.p. = 250–254 °C; UV-Vis (MeOH): λ_max_ (log ε) = 583 nm (4.58); IR (ATR): ν = 2940*m*, 1739*m*, 1595*m*, 1494*m*, 1297*vs*, 1252*s*, 1187*s*, 1099*s*, 1033*s*, 623*w*, 421*s*, cm^−1^; ^1^H NMR (500 MHz, CDCl_3_): δ = 7.69–7.64 (*m*, 2H, 44-H + 45-H), 7.52–7.47 (*m*, 1H, 46-H), 7.31–7.27 (*m*, 1H, 43-H), 6.69–6.63 (*m*, 2H, 49-H), 5.31–5.25 (*m*, 1H, 2-H), 4.56 (*d*, *J* = 3.6 Hz, 1H, 3-H), 3.58–3.44 (*m*, 8H, 56-H, 57-H), 3.21–3.09 (*m*, 1H, 19-H), 3.10–2.95 (*m*, 12H, 34-H, 35-H, 36-H, 37-H, 54-H), 2.78–2.49 (*m*, 5H, 59-H, 13-H), 2.13 (*s*, 3H, 29-H), 2.12–1.75 (*m*, 13H, 55-H, 18-H, 16-H_a_, 1-H_a_, 58-H, 22-H_a_, 21-H_a_), 1.99 (*s*, 3H, 31-H), 1.98 (*s*, 3H, 33-H), 1.68–1.07 (*m*, 13H, 16-H_b_, 6-H, 22-H_b_, 21-H_b,_ 7-H, 11-H, 15-H, 9-H, 1-H_b_), 1.07 (*s*, 3H, 25-H), 0.99 (*s*, 3H, 24-H), 1.06–0.88 (*m*, 2H, 12-H), 0.93 (*s*, 3H, 27-H), 0.89 (*s*, 3H, 26-H), 0.91–0.86 (*m*, 1H, 5-H), 0.85 (*s*, 3H, 23-H) ppm; ^13^C NMR (125 MHz, CDCl_3_): δ = 212.9 (C-20), 173.5 (C-28), 170.7 (C-32), 170.3 (C-30), 167.9 (C-40), 152.1 (C-47), 151.4 (C-51), 151.3 (C-53), 134.8 (C-41), 132.0 (C-42), 130.8 (C-43), 130.3 (C-45), 129.9 (C-44), 127.6 (C-46), 126.6 (C-49), 123.7 (C-50), 113.3 (C-48), 105.5 (C-52), 78.1 (C-3), 69.7 (C-2), 55.4 (C-5), 54.5 (C-17), 52.5 (C-18), 51.2 (C-9), 51.1 (C-57), 50.6 (C-56), 50.1 (C-19), 44.5 (C-34, C-35, C-36, C-37), 42.3 (C-14), 42.0 (C-1), 40.8 (C-8), 37.5 (C-4), 37.1 (C-10), 35.9 (C-13), 35.6 (C-22), 34.1 (C-7), 32.1 (C-16), 30.4 (C-29), 29.9 (C-15), 29.0 (C-23), 28.8 (C-21), 27.8 (C-59), 27.5 (C-12), 21.4 (C-11), 21.3 (C-31), 20.9 (C-31), 20.7 (C-58), 20.0 (C-54), 19.7 (C-55), 18.1 (C-6), 17.6 (C-24), 16.8 (C-25), 16.2 (C-26), 14.6 (C-27) ppm; MS (ESI, MeOH): *m*/*z* 1100.2 (100%, [M-Cl]^+^); analysis calcd for C_69_H_87_N_4_O_7_Cl (1135.62): C 72.96, H 7.72, N 4.93; found: C 72.69, H 7.97; N 4.76.

#### 4.2.30. 9-[2-[[4-(2β,3β-Diacetoxy-20-oxo-30-norlupan-28-oyl)-1-(hexahydro-1H-1,4-diazepin-1-yl)]carbonyl]phenyl]-3,6-bis(dimethylamino)-xanthylium Chloride (**27**) 

According to the GPC from **12** (90 mg, 0.14 mmol), followed by chromatography (SiO_2_, CHCl_3_/MeOH, 9:1), **27** (90 mg, 63%) was obtained as a purple solid; R_f_ = 0.52 (SiO_2_, CHCl_3_/MeOH, 9:1); m.p. = 265–269 °C; UV-Vis (MeOH): λ_max_ (log ε) = 555 nm (4.93); IR (ATR): ν = 2932*br*, 1737*m*, 1592*vs*, 1493*m*, 1407*s*, 1341*s*, 1251*m*, 1185*vs*, 1132*m*, 1031*w*, 925*m*, 820*w*, 699*m*, 579*w*, 516*w* cm^−1^; ^1^H NMR (500 MHz, CDCl_3_): δ = 7.69–7.48 (*m*, 2H, 43-H, 42-H), 7.47–7.38 (*m*, 1H, 45-H), 7.37–7.29 (*m*, 1H, 44-H), 7.31–7.03 (*m*, 1H, 49-H), 7.00–6.65 (*m*, 2H, 48-H, 51-H), 5.29–5.27 (*m*, 1H, 2-H), 4.59–4.55 (*m*, 1H, 3-H), 3.38–3.30 (*m*, 22H, 34-H, 35-H, 36-H, 37-H, 38-H, 53-H), 3.24–3.20 (*m*, 1H, 19-H), 2.71–2.61 (*m*, 1H, 13-H), 2.14 (*s*, 4H, 29-H, 16-H_a_), 2.01 (*s*, 3H, 33-H), 2.00–1.99 (*m*, 4H, 31-H, 18-H, 1-H_a_), 1.96–1.90 (*m*, 1H, 22-H_a_), 1.88–1.76 (*m*, 1H, 21-H_a_), 1.64–1.17 (*m*, 12H, 16-H_b_, 6-H, 21-H_b_, 22-H_b_, 7-H, 11-H, 15-H_a_, 1-Hb, 9-H), 1.08 (*s*, 4H, 26-H, 15-H_b_), 1.00 (*s*, 5H, 25-H, 12-H), 0.94 (*s*, 3H, 27-H), 0.92 (*s*, 4H, 24-H, 5-H), 0.85 (*s*, 3H, 23-H) ppm; ^13^C NMR (126 MHz, CDCl_3_): δ = 212.9 (C-20), 174.5 (C-28), 173.7 (C-39), 170.7 (C-32), 170.2 (C-30), 157.5 (C-52), 157.3 (C-50), 155.5 (C-46), 138.2 (C-41), 137.5 (C-40), 132.0 (C-49), 130.1 (C-44), 129.6 (C-43), 127.7 (C-42), 126.5 (C-45), 114.1 (C-47), 113.7 (C-48), 96.7 (C-51), 77.9 (C-3), 69.6 (C-2), 55.3 (C-5), 54.9 (C-17), 52.9 (C-18), 52.1 (C-36, C-38) 51.1 (C-9), 50.1 (C-19), 47.5 (C-34, C-35), 42.2 (C-1), 42.0 (C-14), 41.3 (C-53), 40.7 (C-8), 37.4 (C-4), 37.0 (C-10), 35.8 (C-13), 35.5 (C-22), 34.1 (C-7), 31.9 (C-16), 30.7 (C-37), 30.2 (C-29), 29.7 (C-15), 28.9 (C-23), 28.7 (C-21), 27.2 (C-12), 21.3 (C-11), 20.8 (C-31), 18.0 (C-6), 17.5 (C-25), 16.7 (C-26), 16.2 (C-24), 14.6 (C-27) ppm; MS (ESI, MeOH): *m*/*z* 1010.3 (100%, [M-Cl]^+^); analysis calcd for C_63_H_83_N_4_O_7_Cl (1043.83): C 72.49, H 8.02, N 7.40; found: C 72.19, H 8.31; N 7.26.

#### 4.2.31. 9-[2-[[4-(2β,3β-Diacetoxy-20-oxo-30-norlupan-28-oyl)-1-(hexahydro-1H-1,4-diazepin-1-yl)]carbonyl]phenyl]-3,6-bis(diethylamino)-xanthylium Chloride (**28**) 

According to the GPC from **12** (400 mg, 0.6 mmol), followed by chromatography (SiO_2_, CHCl_3_/MeOH, 9:1), **28** (501 mg, 76%) was obtained as a purple solid; R_f_ = 0.25 (SiO_2_, CHCl_3_/MeOH, 9:1); m.p. = 250–255 °C; UV-Vis (MeOH): λ_max_ (log ε) = 561 nm (4.85); IR (ATR): ν = 2937*br*, 1738*m*, 1586v*s*, 1528*w*, 1467*m*, 1411*s*, 1335*s*, 1245*s*, 1179*vs*, 1131*m*, 1072*m*, 1073*w*, 1011*m*, 975*w*, 920*w*, 822*w*, 683*m* cm^−1^; ^1^H NMR (500 MHz, CDCl_3_) δ = 7.69–7.58 (*m*, 4H, 44-H, 43-H), 7.46–7.39 (*m*, 2H, 45-H), 7.35–6.87 (*m*, 4H, 42-H, 49-H, 48-H), 6.84–6.69 (*m*, 2H, 51-H), 5.32–5.26 (*m*, 1H, 2-H), 4.60–4.55 (*m*, 1H, 3-H), 3.82–3.46 (*m*, 12H, 34-H, 35-H, 36-H, 37-H, 53-H, 38-H), 3.46–3.11 (*m*, 1H, 19-H), 2.73–2.62 (*m*, 1H, 13-H), 2.18–2.08 (*m*, 4H, 29-H, 16-H_a_), 2.01 (*s*, 3H, 31-H), 2.00 (*s*, 3H, 33-H), 2.08–1.89 (*m*, 3H, 18-H, 1-H_a_, 22-H_a_), 1.90–1.70 (*m*, 1H, 21-H_a_), 1.62–1.18 (*m*, 14H, 16-H_b_, 6-H, 21-H_b_, 22-H_b_, 7-H, 11-H, 38-H, 9-H, 15-H_a_, 1-H_b_), 1.34–1.29 (*m*, 12H, 54-H), 1.09 (*s*, 3H, 25-H), 1.01 (*s*, 3H, 26-H), 0.95 (*s*, 3H, 27-H), 0.93–0.87 (*m*, 1H, 5-H), 0.92 (*s*, 3H, 26-H), 0.86 (*s*, 3H, 23-H) ppm; ^13^C NMR (126 MHz, CDCl_3_) δ = 213.1 (C-20), 179.2 (C-28), 170.8 (C-32), 170.3 (C-30), 168.7 (C-39), 157.9 (C-52), 155.9 (C-46), 155.7 (C-50), 138.0 (C-41), 132.5 (C-49), 130.7 (C-40), 130.4 (C-42), 130.0 (C-43), 129.7 (C-44), 126.6 (C-45), 115.3 (C-48), 115.3 (C-47), 96.3 (C-51), 78.0 (C-3), 70.7 (C-53), 69.8 (C-2), 55.4 (C-5), 55.1 (C-17), 52.9 (C-18), 51.3 (C-9), 50.3 (C-19), 46.4 (C-35), 46.3 (C-34), 42.3 (C-14), 42.1 (C-1), 40.8 (C-8), 37.5 (C-4), 37.1 (C-10), 36.0 (C-13), 35.7 (C-22), 34.3 (C-7), 31.6 (C-16), 30.3 (C-29), 30.0 (C-15), 29.9 (C-38), 29.1 (C-23), 28.6 (C-21), 27.4 (C-12), 21.4 (C-11), 21.4 (C-33), 21.0 (C-31), 18.1 (C-6), 17.6 (C-24), 16.9 (C-25), 16.4 (C-26), 14.7 (C-27), 12.8 (C-54) ppm; MS (ESI, MeOH): *m*/*z* 1066.7 (100%, [M-Cl]^+^); analysis calcd for C_66_H_89_N_4_O_8_Cl (1101.91): C 71.94, H 8.14, N 5.08; found: C 71.69, H 8.34; N 5.31.

#### 4.2.32. 9-[2-[[4-(2β,3β-Diacetoxy-20-oxo-30-norlupan-28-oyl)-1-(hexahydro-1H-1,4-diazepin-1-yl)]carbonyl]phenyl)]-2,3,6,7,12,13,16,17-octahydro-1H,5H,11H,15H-xantheno(2,3,4-ij:5,6,7-i’j’)diquinolizin-18-ium Chloride (**29**)

According to the GPC from **12** (186 mg, 0.29 mmol), followed by chromatography (SiO_2_, CHCl_3_/MeOH, 9:1), **29** (240 mg, 70%) was obtained as a purple solid; R_f_ = 0.3 (SiO_2_, CHCl_3_/MeOH, 9:1); m.p. = 260–267 °C; UV-Vis (MeOH): λ_max_ (log ε) = 578 nm (5.00); IR (ATR): ν = 3373*br*, 2937*w*, 1714*w*, 1593*s*, 1492*s*, 1460*m*, 1361*m*, 1292*vs*, 1265*vs*, 1179*vs*, 1093*s*, 1073*w*, 1035*m*, 894*w*, 772*w*, 730*w*, 507*br*, 420*s* cm^−1^; ^1^H NMR (500 MHz, CDCl_3_): δ = 7.67–7.56 (*m*, 2H, 43-H, 44-H), 7.47–7.35 (*m*, 1H, 45-H), 7.32–7.12 (*m*, 1H, 43-H), 6.75–6.58 (*m*, 2H, 48-H), 5.31–5.26 (*m*, 1H, 2-H), 4.60–4.55 (*m*, 1H, 3-H), 3.77–3.36 (*m*, 8H, 55-H, 56-H), 3.35–3.12 (*m*, 1H, 19-H), 3.09–2.89 (*m*, 4H, 53-H), 2.83–2.58 (*m*, 5H, 58-H, 13-H), 2.14 (*s*, 3H, 29-H), 2.21–1.86 (*m*, 12H, 54-H, 16-H_a_, 18-H, 1-H_a_, 22-H_a_, 57-H), 2.01 (*s*, 3H, 33-H), 1.99 (*s*, 3H, 31-H), 1.85–1.63 (*m*, 1H, 21-H_a_), 1.62–1.12 (*m*, 13H, 16-H_b_, 6-H, 21-H_b_, 22-H_b_, 7-H, 11-H, 15-H, 1-H_b_, 9-H), 1.07 (*s*, 3H, 25-H), 1.00 (*s*, 3H, 24-H), 0.94 (*s*, 3H, 27-H), 0.91 (*s*, 3H, 26-H), 0.90–0.87 (*m*, 1H, 5-H), 0.85 (*s*, 3H, 23-H) ppm; ^13^C NMR (125 MHz, CDCl_3_): δ = 213.1 (C-20), 174.8 (C-28), 170.8 (C-32), 170.3 (C-30), 152.0 (C-46), 151.5 (C-50), 151.4 (C-52), 136.3 (C-40), 131.3 (C-41), 130.5 (C-42), 129.9 (C-44), 129.6 (C-43), 126.9 (C-48), 126.4 (C-45), 123.7 (C-49), 113.0 (C-47), 105.4 (C-51), 78.0 (C-3), 69.7 (C-2), 55.4 (C-5), 55.1 (C-17), 52.9 (C-18), 51.3 (C-9), 51.0 (C-56), 50.6 (C-55), 50.3 (C-19), 42.3 (C-14), 42.1 (C-1), 40.8 (C-8), 37.5 (C-4), 37.1 (C-10), 35.9 (C-13), 35.6 (C-22), 34.3 (C-7), 32.0 (C-16), 30.3 (C-29), 29.8 (C-15), 29.0 (C-23), 28.8 (C-21), 27.6 (C-58), 27.4 (C-12), 21.4 (C-11), 21.3 (C-33), 21.0 (C-31), 20.7 (C-57), 20.0 (C-53), 19.8 (C-54), 18.1 (C-6), 17.6 (C-24), 16.8 (C-25), 16.3 (C-26), 14.7 (C-27) ppm; MS (ESI, MeOH): *m*/*z* 1114.5 (100%, [M-Cl]^+^); analysis calcd for C_70_H_89_N_4_O_8_Cl (1149.95): C 73.11, H 7.80, N 4.87; found: C 72.97, H 8.02; N 4.60.

#### 4.2.33. 9-[2-[[4-(3β-Acetyloxy-lup-20(29)en-28-oyl)-1-piperazinyl]carbonyl]phenyl]-3,6-bis(diethylamino)-xanthylium Chloride (**30**)

Compound **30** was synthesized as previously reported and obtained as a purple solid; yield: 66%; R_f_ = 0.4 (SiO_2_, CHCl_3_/MeOH, 9:1); m.p.: 245–249 °C (lit.: [28] 246–250 °C); MS (ESI, MeOH): *m/z* 991.5 (100%, [M-Cl]^+^).

#### 4.2.34. 9-[2-[[4-[(3β)-Acetyloxy-20(29)en-28-oxo-lup-28-yl]-1-homopiperazinyl]carbonyl]phenyl]-3,6-bis(diethylamino)-xanthylium Chloride (**31**)

Compound **31** was synthesized as previously reported and obtained as a purple solid; yield: 58%; R_f_ = 0.50 (SiO_2_, MeCN/CH_2_Cl_2_/H_2_O, 10:1:1); m.p.: 258–262 °C (lit.: [29] 256–260 °C); MS (ESI, MeOH): *m/z* 1005.6 (100%, [M-Cl]^+^).

#### 4.2.35. 3β-Acetyloxy-28-[4-[3-(2,3,6,7,12,13,16,17-octahydro-1H,5H,11H,15H-pyrido[3,2,1-ij] pyrido[1″,2″,3″:1′,8′]quinolino[6′,5′:5,6]pyrano[2,3-f]quinolin-4-ium-9-yl)benzoyl]piperazine-1-yl]-28-oxo-lup-20(29)-en chloride (**32**)

Compound **32** was synthesized as previously reported and obtained as a purple solid; yield: 52%; R_f_ = 0.38 (SiO_2_, CHCl_3_/MeOH, 9:1); m.p.: >300 °C (lit: [24] > 300 °C); MS (ESI, MeOH): *m/z* 1039.4 (100%, [M-Cl]^+^).

#### 4.2.36. 3β-Acetyloxy-28-[4-[3-(2,3,6,7,12,13,16,17-octahydro-1H,5H,11H,15H-pyrido[3,2,1-ij] pyrido[1″,2″,3″:1′,8′]quinolino[6′,5′:5,6]pyrano[2,3-f]quinolin-4-ium-9-yl)benzoyl]homopiperazine-1-yl]-28-oxo-lup-20(29)-en Chloride (**33**)

Compound **33** was synthesized as previously reported and obtained as a purple solid; yield: 68%; R_f_ = 0.38 (SiO_2_, CHCl_3_/MeOH, 9:1); m.p.: >300 °C (lit: [24] > 300 °C); MS (ESI, MeOH): *m/z* 1052.8 (100%, [M-Cl]^+^).

#### 4.2.37. 9-[2-[[4-(3β-Acetyloxy-20-oxo-norlupan-28-oyl)-1-piperazinyl]carbonyl]phenyl]-3,6-bis(diethylamino)-xanthylium Chloride (**34**)

Compound **34** was synthesized as previously reported and obtained as a purple solid; yield: 70%; R_f_ = 0.38 (SiO_2_, CHCl_3_/MeOH, 9:1); m.p.: 236–242 °C (lit.: [28] 235–243 °C); MS (ESI, MeOH): *m/z* 993.6 (100%, [M-Cl]^+^).

#### 4.2.38. 9-[2-[[4-(3β-Acetyloxy-20,28-dioxo-30-norlupan-28-yl)-1-homopiperazinyl]carbonyl]phenyl]-3,6-bis(diethylamino)-xanthylium Chloride (**35**)

Compound **35** was synthesized as previously reported and obtained as a purple solid; yield: 87%; R_f_ = 0.33 (SiO_2_, CHCl_3_/MeOH, 9:1); m.p.: 245–248 °C (lit.: [24] 248–250 °C); MS (ESI, MeOH): *m/z* 1007.64 (100%, [M-Cl]^+^).

#### 4.2.39. 3β-Acetyloxy-28-[4-[3-(2,3,6,7,12,13,16,17-octahydro-1H,5H,11H,15H-pyrido[3,2,1-ij]pyrido[1″,2″,3″:1′,8′]quinolino[6′,5′:5,6]pyrano[2,3-f]quinolin-4-ium-9-yl)benzoyl]piperazine-1-yl]-20,28-dioxo-30-norlupan-12-en Chloride (**36**)

Compound **36** was synthesized as previously reported and obtained as a purple solid; yield: 65%; R_f_ = 0.45 (SiO_2_, CHCl_3_/MeOH, 9:1); m.p.: >300 °C (lit.: [24] > 300 °C); MS (ESI, MeOH): *m/z* 1041.5 (100%, [M-Cl]^+^).

#### 4.2.40. 3β-Acetyloxy-28-[4-[3-(2,3,6,7,12,13,16,17-octahydro-1H,5H,11H,15H-pyrido[3,2,1-ij]pyrido[1″,2″,3″:1′,8′]quinolino[6′,5′:5,6]pyrano[2,3-f]quinolin-4-ium-9-yl)benzoyl]homopiperazine-1-yl]-20,28-dioxo-30-norlupan-12-en Chloride (**37**)

Compound **37** was synthesized as previously reported and obtained as a purple solid; yield: 66%; R_f_ = 0.45 (SiO_2_, CHCl_3_/MeOH, 9:1); m.p.: > 300 °C (lit.: [24] > 300 °C); MS (ESI, MeOH): *m/z* 1055.2 (100%, [M-Cl]^+^).

#### 4.2.41. (3β, 20R)-Dihydroxy-30-norlupan-28-oic Acid (**38**)

To a solution of **PA** (4.7 g, 10 mmol) in THF (200 mL) and methanol (150 mL), NaBH_4_ (2.64 g, 70.0 mmol) was added in several portions, and the mixture was stirred at 21 °C for 3 days. The usual aq. work-up, followed by chromatography (SiO_2_, hexanes/EtOAc, EtOAc: 10% → 30%) gave **38** (2.70 g, 59%) as a colorless solid; R_f_ = 0.38 (SiO_2_, hexanes/ethyl acetate, 6:4); m.p. = 289–292 °C; ${\left[\alpha \right]}_{D}^{20}$ = −37.27° (*c* 0.134, MeOH); IR (ATR): ν = 3452*m*, 2940*s*, 2666*m*, 1698*s*, 1673*s*, 1629*w*, 1452*m*, 1377*m*, 1359*w*, 1320*w*, 1291*w*, 1277*w*, 1233*w*, 1182*s*, 1131*m*, 1105*m*, 1078*w*, 1032*s*, 984*m*, 973*w*, 945*w*, 921w, 977*w*, 853*m*, 798*w*, 749*w*, 484*m*, 453*m* cm^−1^;^1^H-NMR (500 MHz, CD_3_OD): δ = 3.82 (*q*, *J* = 6.3 Hz, 1H, 20-H), 3.14 (*dd*, *J* = 11.4, 4.9 Hz, 1H, 3-H), 2.31 (*td*, *J* = 12.1, 3.6 Hz, 1H, 13-H), 2.25–2.14 (*m*, 2H, 16-H_a_, 19-H), 1.81–1.68 (*m*, 3H, 22-H_a_, 21-H_a_, 1-H_a_), 1.68–1.48 (*m*, 8H, 18-H, 11-H, 21-H_b_, 12-H_a_, 2-H_a_, 6-H_a_, 15-H_a_), 1.48–1.20 (*m*, 8H, 6-H_b_, 7-H, 16-H_b_, 22-H_b_, 9-H, 2-H_b_, 12-H_b_), 1.16 (*d*, *J* = 13.5 Hz, 1H, 15-H_b_), 1.11 (*d*, *J* = 6.4 Hz, 3H, 29-H), 1.01 (*s*, 3H, 27-H), 0.97 (*s*, 3H, 25-H), 0.96 (*s*, 3H, 23-H), 0.95–0.90 (*m*, 1H, 1-H_b_), 0.87 (*s*, 3H, 26-H), 0.76 (*s*, 3H, 24-H), 0.72 (*d*, *J* = 9.8 Hz, 1H, 5-H) ppm; ^13^C-NMR (126 MHz, CD3OD): δ = 179.04 (C-28), 78.26 (C-3), 68.22 (C-20), 56.51 (C-17), 55.45 (C-5), 50.37 (C-9), 47.37 (C-18), 45.74 (C-19), 42.26 (C-14), 40.50 (C-8), 38.70 (C-1), 38.54 (C-4), 38.11 (C-13), 36.92 (C-10), 36.86 (C-22), 34.25 (C-7), 31.53 (C-16), 29.53 (C-15), 27.21 (C-23), 26.96 (C-12), 26.63 (C-11), 21.97 (C-21), 21.82 (C-29), 20.79 (C-2), 18.05 (C-6), 15.28 (C-25), 15.28 (C-26), 14.71 (C-24), 13.70 (C-27) ppm; MS (ESI, MeOH): *m*/*z* = 559.1 (100%, [M-H]^−^), analysis calcd for C_29_H_48_O_4_ (460.36): C 75.61, H 10.50; found: C 72.51, H 9.91.

#### 4.2.42. (3β, 20R)-Bis(acetyloxy)-30-norlupan-28-oic Acid (**39**)

Acetylation of **38** (4.0 g, 8.7 mmol) according to the GPA, followed by chromatography (SiO_2_, CHCl_3_/MeOH, 9:1) gave **39** (3.89 g, 82%) as a colorless solid; R_f_ = 0.50 (SiO_2_, hexanes/ethyl acetate, 7:3); m.p. = 275–277 °C; ${\left[\alpha \right]}_{D}^{20}$ = −22.7° (*c* 0.109, CHCl_3_); IR (ATR): ν = 2946*m*, 1736*s*, 1684*m*, 1454*w*, 1372*m*, 1243*vs*, 1136*w*, 1023*m*, 980*w*, 949*w*, 608*w* cm^−1^; ^1^H NMR (500 MHz, CDCl_3_): δ = 5.05 (*q*, *J* = 6.0 Hz, 1H, 20-H), 4.48 (*dd*, *J* = 11.2, 5.1 Hz, 1H, 3-H), 2.34–2.22 (*m*, 2H, 19-H, 16-H_a_), 2.20–2.10 (*m*, 1H, 13-H), 2.05 (*s*, 3H, 31-H), 2.04 (*s*, 3H, 33-H), 1.90 (*dd*, *J* = 12.3, 7.2 Hz, 1H, 22-H_a_), 1.87–1.72 (*m*, 2H, 21-H), 1.71–1.68 (*m*, 1H, 1-H_a_), 1.68–1.55 (*m*, 3H, 2-H, 12-H_a_), 1.54–1.45 (*m*, 3H, 6-H_a_, 11-H_a_, 15-H_a_), 1.44–1.23 (*m*, 9H, 6-H_b_, 7-H, 22-H_b_, 16-H_b_, 12-H_b_, 11-H_b_, 18-H, 9-H), 1.17 (*d*, *J* = 6.4 Hz, 3H, 29-H), 1.16 –1.13 (*m*, 1H, 15-H_b_), 1.02–0.94 (*m*, 1H, 1-H_b_), 0.91 (*s*, 3H, 25-H), 0.87 (*s*, 3H, 27-H), 0.85 (*s*, 3H, 26-H), 0.84 (*s*, 3H, 24-H), 0.82 (*s*, 3H, 23-H), 0.78 (*d*, *J* = 10.1 Hz, 1H, 5-H) ppm; ^13^C NMR (125 MHz, CDCl_3_): δ = 181.8 (C-28), 171.1 (C-32), 171.1 (C-35), 81.0 (C-3), 77.4, 77.2, 76.9, 72.6 (C-20), 56.7 (C-17), 55.5 (C-5), 50.1 (C-9), 48.1 (C-18), 44.2 (C-19), 42.5 (C-14), 40.8 (C-8), 38.5 (C-1), 38.1 (C-13), 38.0 (C-4), 37.3 (C-22), 36.8 (C-10), 34.4 (C-7), 32.0 (C-16), 29.8 (C-15), 28.1 (C-23), 26.7 (C-12), 23.8 (C-2), 23.7 (C-21), 21.5 (C-33), 21.4 (C-31), 20.9 (C-11), 19.9 (C-29), 18.3 (C-6), 16.7 (C-24), 16.3 (C-25), 16.2 (C-26), 14.5 (C-27) ppm; MS (ESI, MeOH/CHCl_3_): *m*/*z =* 567.1 (75%, [M+Na]^+^), 1113.3 (100%, [2M+Na]^+^); analysis calcd for C_35_H_52_O_6_ (544.77): C 72.76, H 9.62; found: C 72.51, H 9.91.

#### 4.2.43. (3β, 20R)-Bis(acetyloxy)-28-(1-piperazinyl)-30-norlupan-28-one (**40**)

According to the GPB from **39** (1.0 g, 1.8 mmol), followed by chromatography (SiO_2_, CHCl_3_/MeOH, 9:1), **40** (922 mg, 82%) was obtained as a colorless solid; R_f_ = 0.6 (SiO_2_, CHCl_3_/MeOH, 9:1); m.p. = 180–183 °C; ${\left[\alpha \right]}_{D}^{20}$ = −14.2° (*c* 0.049, CHCl_3_); IR (ATR): ν = 2941*m*, 1734*s*, 1633*m*, 1455*w*, 1370*m*, 1244*vs*, 1190*m*, 1134*w*, 1008*m* cm^−1^; ^1^H NMR (500 MHz, CDCl_3_): δ = 5.04 (*d*, *J* = 6.1 Hz, 1H; 20-H), 4.47 (*dd*, *J* = 10.8, 5.5 Hz, 1H, 3-H), 3.73–3.50 (*m*, 4H, 34-H, 36-H), 3.30–3.09 (*m*, 4H, 37-H, 38-H), 2.90–2.81 (*m*, 1H, 13-H), 2.28–2.15 (*m*, 1H, 19-H), 2.11–2.04 (*m*, 1H, 16-H_a_), 2.03 (*s*, 6H, 31-H, 33-H), 1.92–1.75 (*m*, 2H, 22-H_a_, 21-H_a_), 1.72–1.64 (*m*, 1H, 1-H_a_), 1.64–1.43 (*m*, 8H, 2-H, 12-H_a_, 21-H_b_, 6-H, 11-H_a_, 16-H_b_), 1.41–1.17 (*m*, 7H, 7-H, 22-H_b_, 15-H_a_, 11-H_b_, 18-H, 9-H, 12-H_b_), 1.16–1.12 (*m*, 3H, 29-H), 1.11–1.08 (*m*, 1H, 15-H_b_), 0.98–0.94 (*m*, 1H, 1-H_b_), 0.92–0.89 (*m*, 3H, 25-H), 0.85 (*s*, 3H, 27-H), 0.84 (*s*, 3H, 26-H), 0.83 (*s*, 3H, 24-H), 0.82 (*s*, 3H, 23-H), 0.80–0.74 (*m*, 1H, 5-H) ppm; ^13^C NMR (125 MHz, CDCl_3_): δ = 173.8 (C-28), 171.1 (C-30), 171.0 (C-32), 81.0 (C-3), 73.1 (C-20), 55.6 (C-5), 55.0 (C-17), 51.6 (C-9), 50.4 (C-18), 46.3 (C-37, C-41), 43.8 (C-38, C-40), 43.0 (C-19), 41.9 (C-14), 40.8 (C-8), 38.5 (C-1), 38.0 (C-4), 37.3 (C-10), 36.5 (C-13), 36.1 (C-22), 34.5 (C-7), 32.4 (C-16), 29.9 (C-15), 28.1 (C-23), 27.0 (C-12), 24.4 (C-21), 23.8 (C-2), 21.5 (C-33), 21.4 (C-31), 21.2 (C-11), 19.9 (C-29), 18.3 (C-6), 16.7 (C-24), 16.3 (C-25), 16.2 (C-26), 14.4 (C-27) ppm; MS (ESI, MeOH/CHCl_3_): *m*/*z* 614 (100%, [M+H]^+^), 636.1 (60%, [M+Na]^+^), 1227.4 (60%, [2M+H]^+^); analysis calcd for C_37_H_60_N_2_O_5_ (612.90): C 72.51, H 9.87, N 4.57; found: C 72.30, H 10.03; N 4.29.

#### 4.2.44. (3β, 20R)-Bis(acetyloxy)-28-(hexahydro-1H-1,4-diazepin1-yl)-30-norlupan-28-one (**41**)

According to the GPB from **39** (1.0 g, 1.8 mmol), followed by chromatography (SiO_2_, CHCl_3_/MeOH, 9:1), **41** (813 mg, 72%) was obtained as a colorless solid; R_f_ = 0.57 (SiO_2_, CHCl_3_/MeOH, 9:1); m.p. = 158–162 °C; ${\left[\alpha \right]}_{D}^{20}$ = −27.5° (*c* 0.094, CHCl_3_); IR (ATR): ν = 2940*m*, 1732*s*, 1624*m*, 1455*w*, 1370*m*, 1243*vs*, 1189*w*, 1134*w*, 1024*m*, 979*w*, 607*w* cm^−1^; ^1^H NMR (500 MHz, CDCl_3_): δ = 5.05–4.99 (*m*, 1H, 20-H), 4.46 (*dd*, *J* = 10.8, 5.5 Hz, 1H, 3-H), 3.78–3.57 (*m*, 10H, 34-H, 35-H, 36-H, 37-H, 38-H), 2.96–2.83 (*m*, 1H, 13-H), 2.28–2.20 (*m*, 1H, 19-H), 2.15–2.08 (*m*, 1H, 16-H_a_), 2.03 (*s*, 3H, 31-H), 2.02 (*s*, 3H, 33-H), 1.93–1.73 (*m*, 2H, 22-H_a_, 21-H_a_), 1.67 (*d*, *J* = 13.2 Hz, 1H, 1-H_a_), 1.64–1.40 (*m*, 7H, 2-H, 12-H_a_, 21-H_b_, 11-H_a_, 6-H, 16-H_b_), 1.41–1.17 (*m*, 8H, 7-H, 15-H_a_, 11-H_b_, 18-H, 22-H_b_, 12-H_b_, 9-H), 1.15 (*d*, *J* = 6.4 Hz, 3H, 29-H), 1.11–1.08 (*m*, 1H, 15-H_b_), 0.97–0.92 (*m*, 1H, 1-H_b_), 0.91 (*s*, 3H, 25-H), 0.85 (*s*, 3H, 27-H), 0.83 (*s*, 3H, 26-H), 0.82 (*s*, 3H, 24-H), 0.81 (*s*, 3H, 23-H), 0.79–0.74 (*m*, 1H, 5-H) ppm; ^13^C NMR (125 MHz, CDCl_3_): δ = 174.8 (C-28), 171.1 (C-32), 171.0 (C-30), 81.0 (C-3), 73.0 (C-20), 55.6 (C-5), 55.4 (C-17), 51.9 (C-9), 50.5 (C-18), 46.9 (C-34, C-35, C-36, C-37, C-38), 43.0 (C-19), 42.0 (C-8), 40.8 (C-14), 38.5 (C-1), 38.0 (C-4), 37.3 (C-10), 36.5 (C-13), 36.3 (C-22), 34.5 (C-7), 32.1 (C-16), 30.0 (C-15), 28.1 (C-23), 26.9 (C-12), 24.5 (C-21), 23.8 (C-2), 21.5 (C-33), 21.4 (C-31), 21.2 (C-11), 19.9 (C-29), 18.3 (C-6), 16.6 (C-24), 16.3 (C-25), 16.2 (C-26), 14.4 (C-27) ppm; MS (ESI, MeOH/CHCl_3_): *m*/*z* 627.6 (70%, [M-OAc+H]^+^), 685 (15%, [M+H]^+^); analysis calcd for C_40_H_64_N_2_O_7_ (684.96): C 70.14, H 9.42, N 4.09; found: C 69.88, H 9.64; N 3.81.

#### 4.2.45. 9-[2-[[4-(2β,20-Diacetoxy-30-norlupan-28-oyl)-1-homopiperazinyl]carbonyl]phenyl]-3,6-bis(dipropylamino)-xanthylium Chloride (**42**)

According to the GPC from **41** (175 mg, 0.28 mmol), followed by chromatography (SiO_2_, CHCl_3_/MeOH, 9:1), **42** (202 mg, 64%) was obtained as a purple solid; R_f_ = 0.48 (SiO_2_, CHCl_3_/MeOH, 9:1); m.p. = 217–221 °C; UV-Vis (MeOH): λ_max_ (log ε) = 566 nm (5.01); IR (ATR): 2935*br*, 1730*m*, 1625*m*, 1587*vs*, 1527*w*, 1468*m*, 1412*m*, 1338*s*, 1300*w*, 1231*s*, 1178*s*, 1133*m*, 1100*m*, 940*w*, 823*w*, 750*w*, 706*w*, 508*w* cm^−1^; ^1^H NMR (500 MHz, CDCl_3_) δ = 7.70–7.56 (*m*, 2H, 42-H, 43-H), 7.47–7.39 (*m*, 1H, 45-H), 7.36–7.15 (*m*, 2H, 44-H, 49-H), 7.05–6.65 (*m*, 2H, 48-H, 51-H), 5.03–4.95 (*m*, 1H, 20-H), 4.48–4.41 (*m*, 1H, 3-H), 3.88–3.23 (*m*, 13H, 34-H, 35-H, 36-H, 37-H, 38-H, 53-H), 2.94–1.79 (*m*, 1H, 13-H), 2.35–2.14 (*m*, 2H, 19-H, 16-H_a_), 2.01 (*s*, 3H, 33-H), 1.99 (*s*, 3H, 31-H), 1.97–1.94 (*m*, 1H, 22-H_a_), 1.91–1.64 (*m*, 4H, 21-H_a_, 15-H_a_, 54-H), 1.64–1.49 (*m*, 5H, 1-H_a_, 2-H, 12-H_a_, 21-H_b_), 1.50–1.30 (*m*, 7H, 6-H, 11-H, 16-H_b_, 7-H), 1.30–1.17 (*m*, 5H, 15-H_b_, 22-H_b_, 18-H, 12-H_b_, 9-H), 1.18–1.11 (*m*, 3H, 29-H), 1.05–0.96 (*m*, 3H, 55-H), 0.95–0.89 (*m*, 1H, 1-H_b_), 0.88–0.72 (*m*, 16H, 27-H, 26-H, 25-H, 24-H, 23-H, 5-H) ppm; ^13^C NMR (126 MHz, CDCl_3_): δ = 174.1 (C-28), 173.7 (C-39), 171.1 (C-30), 171.0 (C-32), 157.7 (C-52), 156.3 (C-50), 156.2 (C-46), 138.2 (C-41), 137.5 (C-40), 132.5 (C-49), 130.3 (C-44), 130.1 (C-43), 129.7 (C-42), 126.8 (C-45), 114.0 (C-47), 113.5 (C-48), 96.5 (C-51), 81.0 (C-3), 73.1 (C-20), 55.5 (C-5), 55.4 (C-17), 53.9 (C-53), 53.9 (C-34, C-35, C-36, C-37, C-38), 52.1 (C-9), 50.4 (C-18), 43.1 (C-19), 42.0 (C-14), 40.8 (C-8), 38.4 (C-1), 37.9 (C-4), 37.2 (C-10), 36.4 (C-13), 36.3 (C-22), 34.5 (C-7), 31.9 (C-16), 29.8 (C-15), 28.0 (C-23), 27.0 (C-12), 24.6 (C-21), 23.8 (C-2), 21.4 (C-31, C-33), 21.2 (C-11), 20.9 (C-54), 19.9 (C-29), 18.3 (C-6), 17.6 (C-24), 16.6 (C-25), 16.3 (C-26), 13.6 (C-27), 11.4 (C-55) ppm; MS (ESI, MeOH): *m*/*z* 1108.3 (100%, [M-Cl]^+^); analysis calcd for C_70_H_99_N_4_O_7_Cl (1144.72): C 73.49, H 8.72, N 4.90; found: C 73.21, H 8.90; N 4.77.

#### 4.2.46. 9-[2-[[4-(2β,20-Diacetoxy-30-norlupan-28-oyl)-1-piperazinyl]carbonyl]phenyl]-3,6-bis(dibutylamino)-xanthylium Chloride (**43**)

According to the GPC from **40** (200 mg, 0.28 mmol), followed by chromatography (SiO_2_, CHCl_3_/MeOH, 9:1), **43** (240 mg, 60%) was obtained as a purple solid; R_f_ = 0.52 (SiO_2_, CHCl_3_/MeOH, 9:1); m.p. = 204–207 °C; UV-Vis (MeOH): λ_max_ (log ε) = 567 nm (5.02); IR (ATR): ν = 2931*br*, 1730*m*, 1633*m*, 1587*vs*, 1528*w*, 1461*m*, 1411*m*, 1339*s*, 1289*m*, 1248*s*, 1218*s*, 1175*s*, 1132*m*, 1109*m*, 1002*m*, 921*w*, 822*w*, 749*w*, 704*w*, 509*w* cm^−1^; ^1^H NMR (500 MHz, CDCl_3_): δ = 7.71–7.61 (*m*, 2H, 42-H, 43-H), 7.56–7.46 (*m*, 1H, 44-H), 7.37–7.31 (*m*, 1H, 41-H), 7.31–7.19 (*m*, 1H, 45-H), 7.02–6.67 (*m*, 2H, 47-H, 50-H), 4.99 (*d*, *J* = 6.2 Hz, 1H, 20-H), 4.44 (*dd*, *J* = 11.0, 5.1 Hz, 1H, 3-H), 3.65–3.21 (*m*, 10H, 34-H, 35-H, 36-H, 37-H, 52-H), 2.78–2.67 (*m*, 1H, 13-H), 2.22–2.11 (*m*, 1H, 19-H), 2.01 (*s*, 3H, 33-H), 2.00 (*s*, 3H, 31-H), 1.99–1.98 (*m*, 1H, 16-H_a_), 1.85–1.72 (*m*, 3H, 22-H_a_, 21-H_a_, 15-H_a_), 1.68–1.62 (*m*, 3H, 53-H, 1-H_a_), 1.61–1.56 (*m*, 2H, 2-H), 1.56–1.50 (*m*, 1H, 12-H_a_), 1.47–1.36 (*m*, 6H, 54-H, 6-H, 21-H_b_, 16-H_b_), 1.36–1.13 (*m*, 8H, 7-H, 11-H, 22-H_b_, 18-H, 9-H, 15-H_b_, 12-H_b_), 1.09 (*d*, *J* = 6.4 Hz, 3H, 29-H), 0.97 (*t*, *J* = 7.1 Hz, 3H, 55-H), 0.93–0.89 (*m*, 1H, 1-H_b_), 0.87–0.82 (*m*, 6H, 27-H, 25-H), 0.82–0.77 (*m*, 9H, 26-H, 24-H, 23-H), 0.76–0.71 (*m*, 1H, 5-H) ppm; ^13^C NMR (126 MHz, CDCl_3_): δ = 174.4 (C-28), 171.0 (C-32), 171.0 (C-30), 167.8 (C-38), 157.8 (C-49), 156.1 (C-51), 156.0 (C-45), 135.1 (C-40), 132.3 (C-48), 130.7 (C-39), 130.5 (C-42), 130.4 (C-41), 130.3 (C-43), 127.7 (C-44), 114.5 (C-47), 114.0 (C-46), 96.5 (C-50), 81.0 (C-3), 72.8 (C-20), 55.5 (C-5), 55.0 (C-17), 52.1 (C-34, C-35, C-36, C-37, C-52), 51.5 (C-9), 50.3 (C-18), 42.9 (C-19), 41.9 (C-8), 40.7 (C-14), 38.4 (C-1), 37.9 (C-4), 37.2 (C-10), 36.4 (C-13), 36.0 (C-22), 34.4 (C-7), 32.3 (C-16), 29.8 (C-15), 29.8 (C-53), 28.0 (C-23), 26.9 (C-12), 24.3 (C-21), 23.8 (C-2), 21.4 (C-33), 21.4 (C-31), 21.1 (C-11), 20.3 (C-54), 19.8 (C-29), 18.3 (C-6), 16.6 (C-24), 16.3 (C-25), 16.2 (C-26), 14.3 (C-27), 14.0 (C-55) ppm; MS (ESI, MeOH): *m*/*z* 1150.4 (100%, [M-Cl]^+^); analysis calcd for C_73_H_105_N_4_O_7_Cl (1186.77): C 73.92, H 8.92, N 4.72; found: C 73.66, H 9.15; N 4.56.

#### 4.2.47. 9-[2-[[4-(2β,20-Diacetoxy-30-norlupan-28-oyl)-1-homopiperazinyl]carbonyl]phenyl]-3,6-bis(dibutylamino)-xanthylium Chloride (**44**)

According to the GPC from **41** (175 mg, 0.28 mmol), followed by chromatography (SiO_2_, CHCl_3_/MeOH, 9:1), **44** (209 mg, 62%) was obtained as a purple solid; R_f_ = 0.55 (SiO_2_, CHCl_3_/MeOH, 9:1); m.p. = 198–201 °C; UV-Vis (MeOH): λ_max_ (log ε) = 568 nm (4.99); IR (ATR): ν = 2932*br*, 1731*m*, 1626*m*, 1587*vs*, 1463*m*, 1411*m*, 1338*s*, 1291*m*, 1246*s*, 1218*s*, 1175*s*, 1132*m*, 1109*m*, 1020*w*, 921*m*, 822*w*, 750*w*, 704*w*, 604*w*, 508*w* cm^−1^; ^1^H NMR (500 MHz, CDCl_3_): δ = 7.68–7.54 (*m*, 2H, 42-H, 43-H), 7.49–7.39 (*m*, 1H, 45-H), 7.38–7.12 (*m*, 2H, 44-H, 49-H), 6.79–6.63 (*m*, 2H, 51-H, 48-H), 5.02–4.97 (*m*, 1H, 20-H), 4.49–4.41 (*m*, 1H, 3-H), 3.86–3.23 (*m*, 12H, 52-H, 34-H, 35-H, 36-H, 37-H, 38-H), 2.96–2.83 (*m*, 1H, 13-H), 2.44–2.20 (*m*, 1H, 19-H), 2.16–2.08 (*m*, 1H, 16-H_a_), 2.02 (*s*, 3H, 33-H), 2.00 (*s*, 3H, 31-H), 1.93–1.84 (*m*, 1H, 22-H_a_), 1.84–1.75 (*m*, 1H, 21-H_a_), 1.74–1.62 (*m*, 4H, 54-H, 15-H_a_, 1-H_a_), 1.63–1.43 (*m*, 4H, 2-H, 21-H_b_,12-H_a_), 1.48–1.38 (*m*, 6H, 6-H, 55-H, 11-H), 1.37–1.19 (*m*, 8H, 16-H_b_, 7-H, 15-H_b_, 22-H_b_, 18-H, 12-H_b_, 9-H), 1.18–1.11 (*m*, 3H, 29-H), 0.98 (*t*, *J* = 7.2 Hz, 3H, 56-H), 0.96–0.91 (*m*, 1H, 1-H_b_), 0.89–0.84 (*m*, 3H, 25-H), 0.84–0.78 (*m*, 13H, 27-H, 24-H, 26-H, 23-H, 5-H) ppm; ^13^C NMR (126 MHz, CDCl_3_): δ = 174.4 (C-28), 171.0 (C-32), 171.0 (C-30), 167.8 (C-39), 157.7 (C-50), 156.1 (C-52), 156.0 (C-46), 135.0 (C-41), 132.3 (C-49), 130.7 (C-40), 130.5 (C-43), 130.4 (C-42), 130.3 (C-44), 127.7 (C-45), 114.5 (C-48), 113.9 (C-47), 96.5 (C-51), 80.9 (C-3), (C-20), 55.5 (C-5), 55.0 (C-17), 53.2 (C-53), 52.0 (C-34, C-35, C-36, C-37, C-38), 51.4 (C-9), 50.3 (C-18), 42.8 (C-19), 41.9 (C-8), 40.7 (C-14), 38.4 (C-1), 37.9 (C-4), 37.2 (C-10), 36.4 (C-13), 36.0 (C-22), 34.4 (C-7), 32.3 (C-16), 30.5 (C-54), 29.7 (C-15), 28.0 (C-23), 26.9 (C-12), 24.3 (C-21), 23.8 (C-2), 21.4 (C-33), 21.4 (C-31), 21.1 (C-11), 20.3 (C-55), 19.8 (C-29), 18.2 (C-6), 16.6 (C-24), 16.2 (C-25), 16.1 (C-26), 14.3 (C-27), 14.0 (C-56) ppm; MS (ESI, MeOH): *m/z* 1164.1 (100%, [M-Cl]^+^); analysis calcd for C_74_H_107_N_4_O_7_Cl (1200.14): C 74.06, H 8.99, N 4.67; found: C 73.86, H 9.14; N 4.51.

## Figures and Tables

**Table 1:** Results from the cytotoxicity assays (SRB; incubation for 72 h); IC_50_ values in μM (each value represents the mean value of three independent experiments each performed in triplicate; confidence interval CI = 95%); used human tumor cell lines: A375 (melanoma), HT29 (colorectal carcinoma), MCF-7 (breast adenocarcinoma), A2780 (ovarian carcinoma), HeLa (cervical carcinoma) and NIH 3T3 (murine fibroblasts, non-malignant). Doxorubicin (**DX**) has been used as positive standard; n.d. not determined; n.s. not soluble under the conditions of the assay.

	A375	HT29	MCF-7	A2780	HeLa	NIH 3T3
**Rh1**	>30	>30	>30	>30	>30	>30
**Rh2**	>30	>30	>30	>30	>30	>30
**Rh3**	8.2 ± 0.2	9.3 ± 0.4	6.4 ± 0.4	6.7 ± 0.7	8 ± 0.7	12 ± 0.7
**Rh4**	4.3 ± 0.1	4.9 ± 0.3	3.08 ± 0.06	3.4 ± 0.1	4.3 ± 0.3	4.3 ± 0.1
**Rh101**	11.2 ± 1.8	18.5 ± 1.8	8.2 ± 0.8	8.0 ± 1.5	11.8 ± 1.1	11.9 ± 1.3
**DX**	n.d.	0.9 ± 0.2	1.1 ± 0.3	0.02 ± 0.01	n.d.	11.9 ± 1.3
**9**	1.58 ± 0.04	1.62 ± 0.09	1.1 ± 0.07	1.52 ± 0.08	1.8 ± 0.2	1.16 ± 0.08
**10**	1.36 ± 0.01	1.8 ± 0.2	1.22 ± 0.04	1.44 ± 0.04	1.9 ± 0.1	1.51 ± 0.07
**11**	2.1 ± 0.2	3.2 ± 0.1	3.1 ± 0.1	3.0 ± 0.1	3.9 ± 0.4	3.3 ± 0.6
**12**	1.8 ± 0.2	2.5 ± 0.2	2.8 ± 0.1	2.5 ± 0.2	3.8 ± 0.6	2.8 ± 0.3
**13**	0.096 ± 0.006	0.12 ± 0.02	0.1 ± 0.04	0.026 ± 0.002	0.08 ± 0.02	0.25 ± 0.02
**14**	0.054 ± 0.002	0.06 ± 0.01	0.058 ± 0.009	0.02 ± 0.003	0.07 ± 0.03	0.16 ± 0.02
**15**	0.1 ± 0.01	0.13 ± 0.03	0.12 ± 0.02	0.041 ± 0.001	0.1 ± 0.02	0.15 ± 0.04
**16**	0.23 ± 0.03	0.49 ± 0.09	0.6 ± 0.1	0.16 ± 0.02	0.34 ± 0.05	0.69 ± 0.08
**17**	0.041 ± 0.003	0.08 ± 0.03	0.1 ± 0.02	0.02 ± 0.002	0.1 ± 0.03	0.21 ± 0.07
**18**	0.087 ± 0.004	0.06 ± 0.01	0.07 ± 0.02	0.023 ± 0.002	0.13 ± 0.02	0.2 ± 0.01
**19**	0.034 ± 0.002	0.028 ± 0.007	0.039 ± 0.007	0.016 ± 0.001	0.05 ± 0.02	0.15 ± 0.03
**20**	0.079 ± 0.01	0.08 ± 0.02	0.11 ± 0.02	0.035 ± 0.007	0.09 ± 0.01	0.24 ± 0.04
**21**	n.s.	n.s.	n.s.	n.s.	n.s.	n.s.
**22**	0.035 ± 0.002	0.08 ± 0.03	0.08 ± 0.01	0.019 ± 0.001	0.06 ± 0.01	0.27 ± 0.05
**23**	0.6 ± 0.04	0.3 ± 0.03	0.18 ± 0.02	0.13 ± 0.02	0.54 ± 0.07	0.69 ± 0.06
**24**	0.1± 0.03	0.1 ± 0.04	0.1 ± 0.03	0.04 ± 0.06	n.d.	0.2 ± 0.06
**25**	0.14 ± 0.01	0.18 ± 0.03	0.09 ± 0.02	0.047 ± 0.003	0.23 ± 0.06	0.24 ± 0.02
**26**	0.13 ± 0.01	0.24 ± 0.04	0.12 ± 0.02	0.057 ± 0.005	0.34 ± 0.05	0.27 ± 0.05
**27**	0.51 ± 0.03	0.5 ± 0.1	0.24 ± 0.05	0.1 ± 0.03	0.71 ± 0.05	1 ± 0.06
**28**	0.1 ± 0.04	0.25 ± 0.04	0.1 ± 0.05	0.05 ± 0.002	n.d.	0.3 ± 0.05
**29**	0.064 ± 0.003	0.15 ± 0.04	0.060 ± 0.009	0.022 ± 0.001	0.20 ± 0.03	0.25 ± 0.03
**30**	0.09 ± 0.01	0.15 ± 0.02	0.08 ± 0.005	0.05 ± 0.004	0.06 ± 0.005	0.21 ± 0.03
**31**	0.17 ± 0.09	0.28 ± 0.01	0.22 ± 0.02	0.22 ± 0.01	0.27 ± 0.05	0.33 ± 0.07
**32**	0.15 ± 0.01	0.25 ± 0.03	0.23 ± 0.02	0.11 ± 0.01	0.20 ± 0.05	0.36 ± 0.05
**33**	0.17 ± 0.05	0.43 ± 0.08	0.22 ± 0.04	0.19 ± 0.04	0.27 ± 0.14	0.56 ± 0.07
**34**	0.08 ± 0.03	0.09 ± 0.02	0.07 ± 0.002	0.036 ± 0.001	0.042 ± 0.002	0.17 ± 0.01
**35**	0.24 ± 0.02	0.30 ± 0.03	0.15 ± 0.05	0.12 ± 0.02	0.11 ± 0.02	0.34 ± 0.06
**36**	0.25 ± 0.04	0.26 ± 0.04	0.17 ± 0.02	0.17 ± 0.02	0.21 ± 0.02	0.26 ± 0.04
**37**	0.20 ± 0.03	0.35 ± 0.05	0.20 ± 0.05	0.17 ± 0.04	0.31 ± 0.15	0.39 ± 0.05
**38**	>20	>20	>20	>20	>20	>20
**39**	15.2 ± 0.7	>20	13.2 ± 0.8	10.1 ± 0.4	13.7 ± 0.9	8.8 ± 1.0
**40**	1.8 ± 0.1	1.3 ± 0.2	2.1 ± 0.2	1.6 ± 0.2	2 ± 0.2	1.5 ± 0.1
**41**	1.7 ± 0.1	1 ± 0.05	2.3 ± 0.2	1.7 ± 0.1	2.1 ± 0.3	1.3 ± 0.1
**42**	0.091 ± 0.004	0.09 ± 0.01	0.11 ± 0.01	0.048 ± 0.001	0.12 ± 0.03	0.19 ± 0.01
**43**	n.s.	n.s.	n.s.	n.s.	n.s.	n.s.
**44**	n.s.	n.s.	n.s.	n.s.	n.s.	n.s.

## Data Availability

The data presented in this study are available on request from the corresponding author.
